# Feedback loop between hypoxia and energy metabolic reprogramming aggravates the radioresistance of cancer cells

**DOI:** 10.1186/s40164-024-00519-1

**Published:** 2024-05-22

**Authors:** Zheng Shi, Cuilan Hu, Xiaogang Zheng, Chao Sun, Qiang Li

**Affiliations:** 1 Institute of Modern Physics, Chinese Academy of Sciences, Lanzhou, China; 2 Key Laboratory of Heavy Ion Radiation Biology and Medicine of Chinese Academy of Sciences, Lanzhou, China; 3 Key Laboratory of Basic Research on Heavy Ion Radiation Application in Medicine, Lanzhou, China; 4 University of Chinese Academy of Sciences, Beijing, China

**Keywords:** Cancer, Radioresistance, Hypoxia, Energy metabolic reprogramming, Feedback loop

## Abstract

Radiotherapy is one of the mainstream approaches for cancer treatment, although the clinical outcomes are limited due to the radioresistance of tumor cells. Hypoxia and metabolic reprogramming are the hallmarks of tumor initiation and progression and are closely linked to radioresistance. Inside a tumor, the rate of angiogenesis lags behind cell proliferation, and the underdevelopment and abnormal functions of blood vessels in some loci result in oxygen deficiency in cancer cells, i.e., hypoxia. This prevents radiation from effectively eliminating the hypoxic cancer cells. Cancer cells switch to glycolysis as the main source of energy, a phenomenon known as the Warburg effect, to sustain their rapid proliferation rates. Therefore, pathways involved in metabolic reprogramming and hypoxia-induced radioresistance are promising intervention targets for cancer treatment. In this review, we discussed the mechanisms and pathways underlying radioresistance due to hypoxia and metabolic reprogramming in detail, including DNA repair, role of cancer stem cells, oxidative stress relief, autophagy regulation, angiogenesis and immune escape. In addition, we proposed the existence of a feedback loop between energy metabolic reprogramming and hypoxia, which is associated with the development and exacerbation of radioresistance in tumors. Simultaneous blockade of this feedback loop and other tumor-specific targets can be an effective approach to overcome radioresistance of cancer cells. This comprehensive overview provides new insights into the mechanisms underlying tumor radiosensitivity and progression.

## Background

Radiotherapy has been one of the most used treatments for cancer for more than a century, with about 60% of cancer patients using radiotherapy as a first-line treatment [1, 2]. It is characterized by better local control rates and fewer side effects than chemotherapy, and is currently the most effective cytotoxic therapy for solid tumors [3]. Nevertheless, the effect of radiotherapy remains ambiguous due to the development of radioresistance [4, 5]. In the presence of ionizing radiation, tumor cells may exhibit epigenetic reprogramming, leading to the emergence of radiation-resistant cell populations and ultimately to tumor recurrence [6, 7].

Neo-angiogenesis is one of the hallmarks of cancer that sustains the rapidly proliferating tumor cells [8]. However, the proliferation rate of the malignant cells often overwhelms that of angiogenesis in solid tumors, resulting in insufficient oxygen supply and hypoxia at some foci due to the abnormal vasculature. Cancer cells can adapt to hypoxia by undergoing metabolic reprogramming [9], resulting in a series of adaptive responses that culminate in enhanced resistance to radiation [10]. In fact, the cellular response to radiotherapy strongly depends on oxygen levels. The free radicals generated in the presence of oxygen exacerbate radiation-induced damage. According to the widely accepted hypothesis of “oxygen fixation”, DNA damage can be easily repaired in the absence of oxygen [11, 12]. Consistent with this, the levels of reactive oxygen species (ROS) are significantly reduced during hypoxia, which decreases radiation-induced DNA damage [13]. Furthermore, hypoxic conditions also activate autophagy, which accelerates the elimination of ROS products and enhances radioresistance [14].

**Fig. 1:**
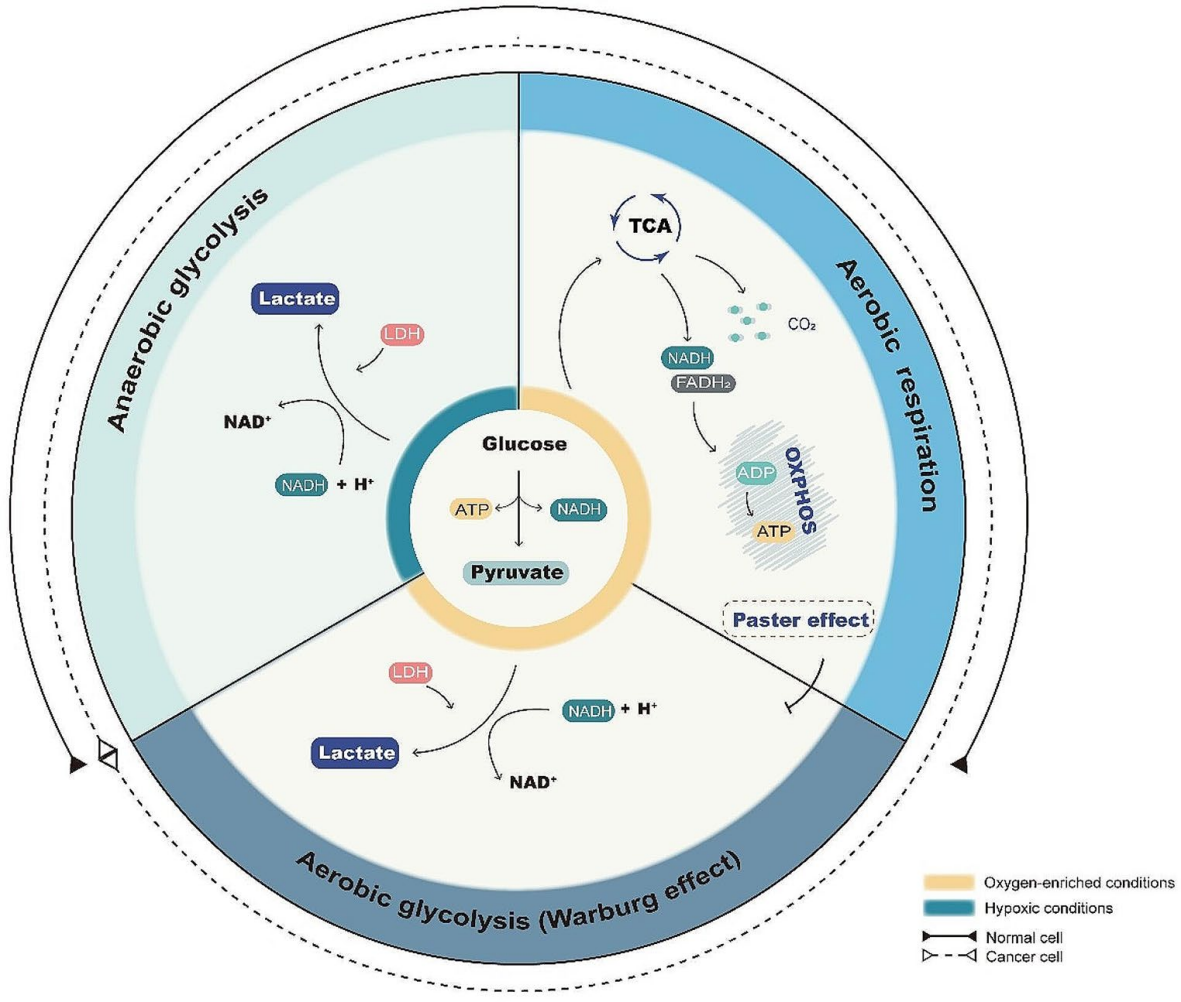
Aerobic glycolysis (Warburg effect), anaerobic glycolysis and aerobic respiration in cells. The yellow area in the figure represents oxygen sufficient conditions and the green represents hypoxia conditions. When oxygen is sufficient, normal cells mainly undergo aerobic respiration instead of glycolysis, as phenomenon known as Paster effect. However, cancer cells produce energy through glycolysis when oxygen is sufficient, which is known as Warburg effect

Glucose is the main source of energy in mammals, and is metabolized into pyruvate via glycolysis. Under aerobic conditions, pyruvate is primarily metabolized through the tricarboxylic acid (TCA) cycle into carbon dioxide and NADH while inhibiting lactate production, a process known as Pasteur effect, NADH is fed into the mitochondrial transport chain to produce ATP through oxidative phosphorylation (OXPHOS). On the other hand, cancer cells produce abundant lactate even under aerobic conditions, a phenomenon known as “aerobic glycolysis”, or “Warburg effect” (Fig. 1). High-throughput glycolysis provides energy, building blocks for biosynthetic pathways, as well as metabolic intermediates for other metabolic pathways [15–17]. It has been shown that phospho-pyruvate dehydrogenase (p-PDH) and pyruvate dehydrogenase kinase 1 (PDK1), which are involved in aerobic glycolysis, are positively associated with radioresistance [18]. Mechanistically, PDK1 inhibits pyruvate metabolism through the TCA cycle via inactivating PDH by phosphorylation [19]. Moreover, PDK1 is associated with multiple targets of AKT, including mTOR, and epithelial-mesenchymal transition (EMT), resulting in radioresistance [20, 21].

Hypoxia is inextricably linked to the metabolic reprogramming of tumor cells [22]. The cells close to the blood vessels mainly produce energy through OXPHOS, while those in the hypoxic regions of the tumor metabolize glucose via the glycolytic pathway. This results in a metabolic symbiosis between the tumor cells in the normoxic and hypoxic areas, which allows the cells to adapt to the complex and hostile environment [23–25]. Therefore, drugs targeting either glycolysis or hypoxia alone cannot reverse radioresistance. Hypoxia-inducible factor-1 (HIF-1) is a key regulator of the cellular response to low oxygen levels [19, 26]. HIF-1 activation is associated with a decrease in ROS levels and increased accumulation of glutathione (GSH), which enhance the radioresistance of cancer cells [9]. Furthermore, HIF-1 also activates glycolysis [27] and the pentose phosphate pathway (PPP) [28], promotes DNA damage repair [29] in cancer cells. In this review, the factors underlying radioresistance induced by hypoxia and metabolic reprogramming, including DNA repair, cancer stem cells (CSCs), oxidative stress relief, autophagy regulation, angiogenesis and immune escape, have been discussed in detail. A greater understanding of these mechanisms can aid in the development of novel radiotherapeutic strategies for treating cancer.

## Mechanisms underlying radioresistance due to hypoxia and metabolic reprogramming

### DNA damage repair

DNA damage caused by radiation mainly includes single-strand break (SSBs), double-strand break (DSBs), base and sugar damage, and cross-linking, of which DSBs are most lethal [30]. The DSBs activate the DNA damage repair (DDR), which allows the cells to recover from radiation-induced damage by inducing cell cycle arrest and DNA repair. There are three main pathways of DNA repair, including the homologous recombination (HR)-based pathway, non-homologous end joining (NHEJ), and alternative end joining, which respond to different types of DNA damage [31]. Inductions of the DDR is one of the main reasons for promoting radioresistance in irradiated cancer cell [32].

Solid tumors usually contain areas of hypoxia. Hypoxia activates AMP-activated protein kinase (AMPK) through activation of LKB1 or CaMKK2, AMPK and HIF exert synergistic protective effects under hypoxic conditions [33]. AMPK promotes radioresistance by activating ataxia telangiectasia-mutated (ATM) and DNA-dependent protein kinase catalytic subunit (DNA-PKcs), which play important roles in DSB repair [34–36]. Lipocalin 2 (LCN2) plays a mediating role in a variety of multiple cachexia-associated diseases [37, 38]. LCN2 was found to be highly expressed in the radioresistant nasopharyngeal carcinoma (NPC) cell line CNE2R, and there was a significant correlation between LCN2 expression and HIF-1α. Knockdown of LCN2 can impair the ability of NPC cells to repair DNA damage or proliferate and enhance the radiosensitivity of NPC cells [39]. Hypoxic exosomes attenuate radiation-induced apoptosis and accelerate DNA damage repair. MiR-340-5p is highly expressed in hypoxic exosomes and is translocated to normoxic cells, inducing radioresistance in oesophageal squamous cell carcinoma (OSCC) cells [40]. All these evidences illustrate the ability of hypoxic conditions to enhance DNA repair of cancer cell.

Much evidence suggests that radioresistance induced by the glycolytic pathway is associated with enhanced DNA damage repair. Elevated glycolysis promotes radiation-induced reattachment of DNA strand breaks through activation of the non-homologous end joining (NHEJ) and homologous recombination (HR) pathways of DSB repair, thereby reducing radiation-induced cytogenetic damage in cells [41]. Research has shown that the glycolytic pathway is strongly associated with radioresistance in prostate cancer. Knockdown of lactate dehydrogenase A (LDHA) or inhibition of LDHA activity can reduce DNA repair capacity [42]. Mucin1 (MUC1), an oncogene overexpressed in many solid tumors, mediates DNA damage repair, and supports glycolysis and nucleotide biosynthesis in cancer cells to enhance DNA repair and radioresistance [43–45]. Apigenin increases radiosensitivity of subcutaneous gliomas in mice by inhibiting NF-κB/HIF-1α-mediated glycolysis and attenuating cell stemness and DNA damage repair [46]. Additionally, heat shock transcription factor 1 (HSF1) are directly involved in the response of tumor cells to hypoxia and acidosis, and promote resistance to chemotherapy and radiotherapy. HSF1 is involved in DNA repair and promotes tumorigenesis through the HSF1-PARP13-PARP1 complex [47]. Cells lacking HSF1 have a reduced ability to repair radiation-induced DSB [48]. Meanwhile, the HSF1/LDHA axis promotes glycolysis that is required for breast cancer cell growth [49] Pyruvate kinase M2 isoform (PKM2) catalyzes the conversion of phosphoenolpyruvate to pyruvate and regulates the final rate-limiting step of glycolysis. It was shown that PKM2-produced pyruvate promotes DNA repair by regulating γH2AX loading to chromatin and establishes a critical role of this mechanism in glioblastoma radioresistance [50]. In addition, it has been revealed that PKM2 is also regulated by hypoxia. It can be activated directly by HIF-1α or indirectly through the HIF-1α/ALYREF/PKM2 axis to promote glycolysis in cancer cells [51]. This double regulation of PKM2 further exacerbates DNA repair and radioresistance.

**Fig. 2:**
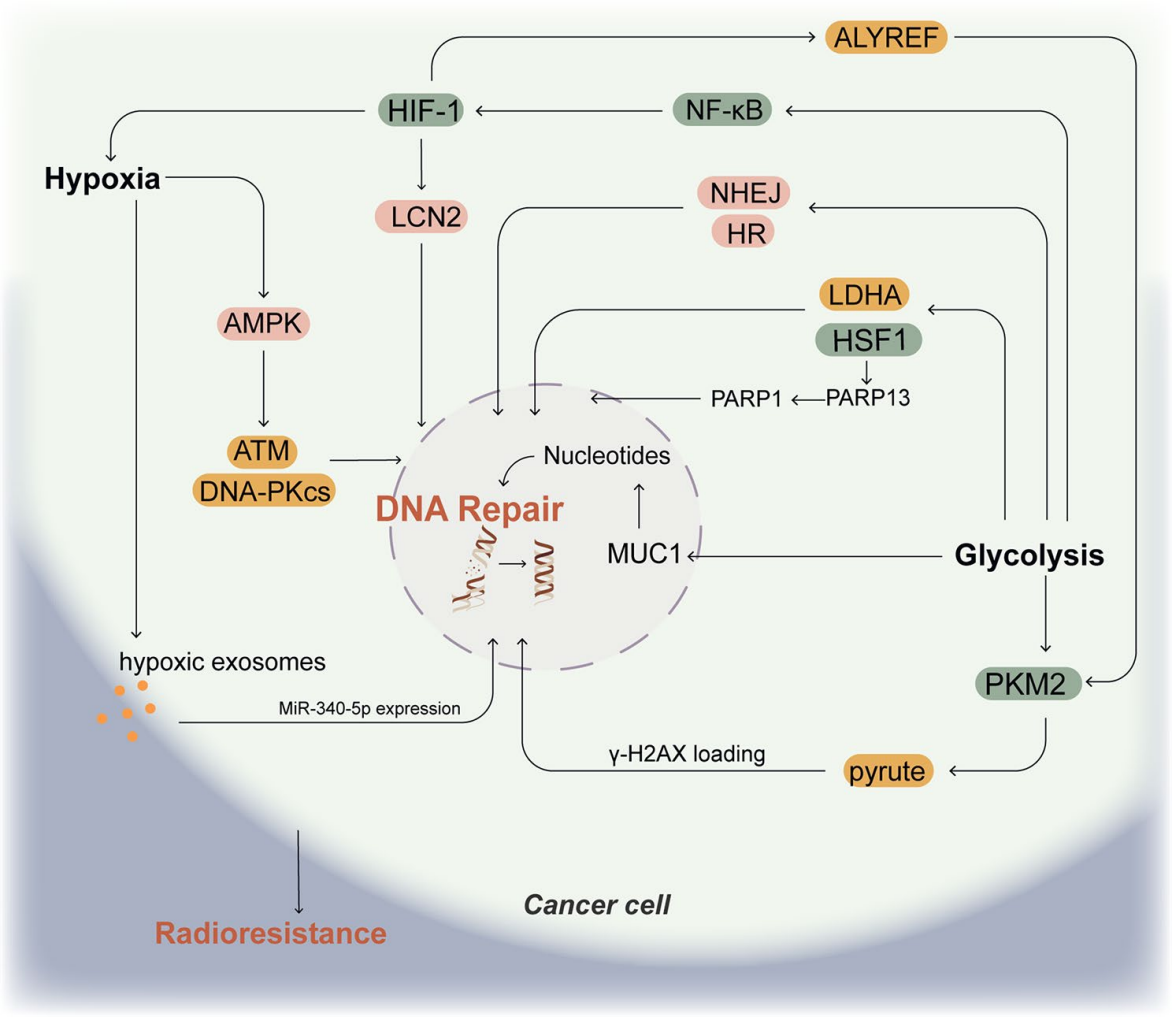
Mechanisms of DNA repair-mediated radioresistance. Radiation-induced cancer cell death is mediated by DNA damage. Hypoxia promotes DNA repair by upregulating HIF-1, AMPK, and the secretion of hypoxic exosomes. Glycolysis upregulates DNA repair through the expression of HSF1, PLKM2, and MUC1 genes. Moreover, glycolysis can also be affected by hypoxia through the HIF-1/NF-κB pathway. Together, these mechanisms exacerbate radioresistance of cancer cell

The mechanisms of DNA repair-mediated radioresistance are depicted Fig. 2. The intermediates of glycolysis flow into many biosynthetic pathways, which generate biomolecules for DNA repair. Given the multiple pathways involved in DNA damage repair in cancer cells, and the pathways overlapping with those in normal cells, it is challenging to identify the suitable therapeutic targets. However, studies appeared that carbon ions are more able to overcome the hypoxia-induced enhancement of DNA repair. In a study of on-small cell lung cancer (NSCLC), carbon ions were found to be effective in scavenging hypoxic tumor cells [52]. Moreover, carbon ions overcame the radioresistance of HNSCC associated with DNA repair, especially in CSCs, and were unaffected by the hypoxic microenvironment, which increased the activation of the NHEJ-c (DNA-PK) and HR pathways (RAD51) only after photon irradiation [53].

### Role of cancer stem cells

CSCs are a rare subpopulation of tumor cells and exhibit self-renewal and multi-lineage differentiation abilities [54, 55]. The origin of CSCs is ambiguous; normal differentiated cells may undergo carcinogenic transformation into stem-like cells [56], and some cancer cells can also dedifferentiate into CSCs with the help of tumor-associated fibroblasts (CAF) [57]. CSCs primarily reside in the nutrient-deficient, hypoxic regions of solid tumors [58]. In addition, exosomes secreted by cancer cells under hypoxic stress are known to induce EMT into CSC-like phenotype [59]. Studies show that CSCs are the seed cells responsible for tumor metastasis and recurrence since they are recalcitrant to surgical resection, radiotherapy or chemotherapy, and may remain latent for many years [56, 60, 61].

**Fig. 3:**
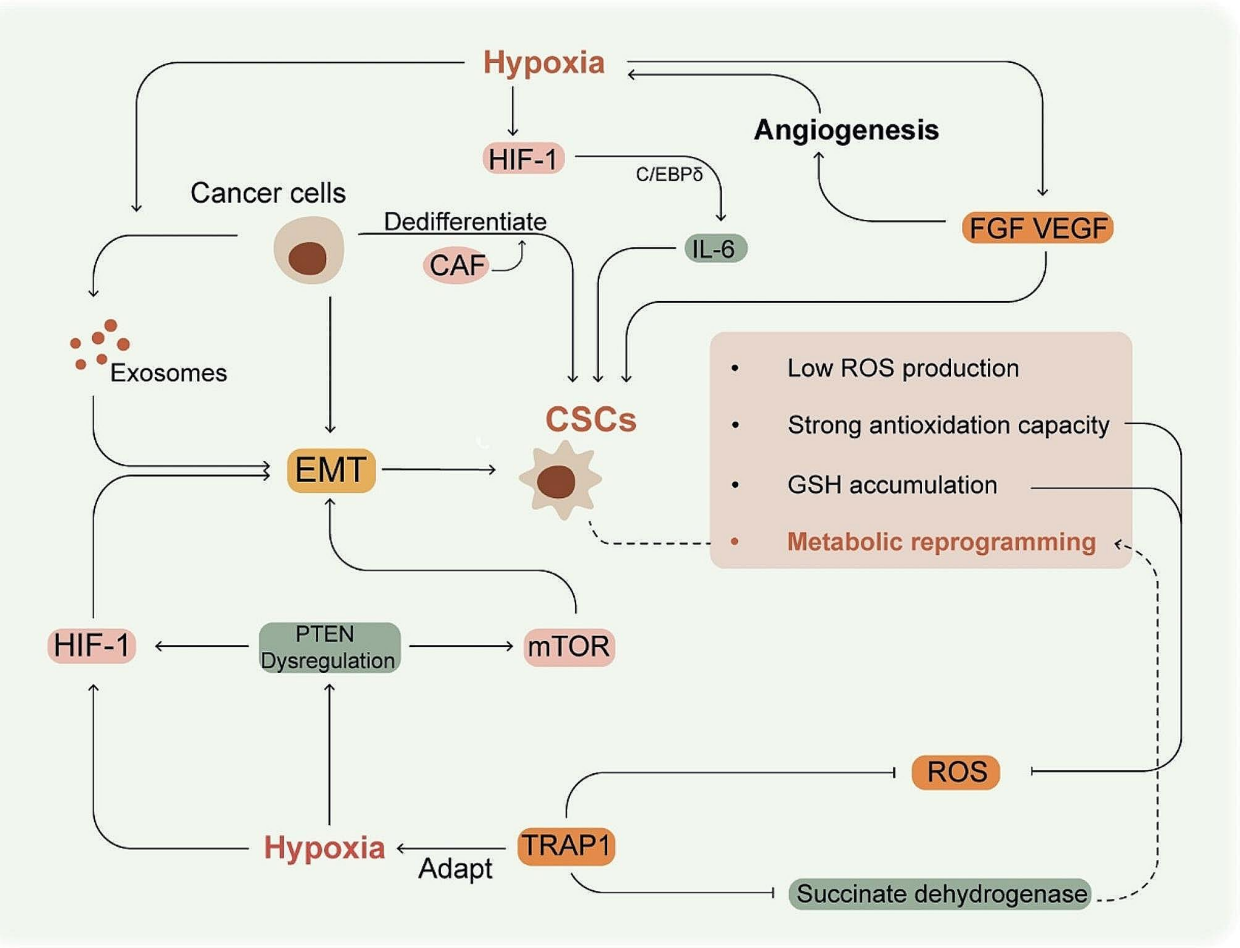
Formation of CSCs under hypoxic conditions. EMT is essential for the formation of CSCs, and is accelerated under hypoxic conditions by increased exosome secretion and HIF-1 expression, and dysregulation of PTEN. CSCs are characterized by low ROS production, strong antioxidant capacity, GSH accumulation and metabolic reprogramming. They suppress ROS levels and adapt to the hypoxic environment, thereby causing further radioresistance. High levels of FGF, VEGF and IL-6 in the tumor microenvironment are hypoxia-regulated and facilitate CSC formation. Moreover, FGF and VEGF are involved in pathological angiogenesis, which exacerbates hypoxia

Low ROS production, metabolic reprogramming, high antioxidant capacity, and GSH accumulation are known to enhance the radioresistance of CSCs [62]. For instance, the radioresistance of breast CSCs (BCSCs) is associated with an increased ability of these cells to scavenge free radicals, which enhances DNA repair [63]. The mechanisms by which CSCs promote radioresistance under hypoxic conditions are shown in Table 1. PTEN dysregulation due to hypoxia also activates HIF-1α and mTOR signaling, resulting in EMT [64]. The activation of HIF-1 in the hypoxic tumor areas induces EMT, resulting in an increase in the number of radioresistant CSCs. Furthermore, HIF-1α mediates the transformation of Hep-2 human laryngeal squamous carcinoma cells to stem-like cells under hypoxic conditions, resulting in increased radioresistance [65]. Many cytokines that are secreted in tumor microenvironment (TME) are hypoxia-regulated and facilitate CSC formation. C/EBPδ links IL-6 and HIF-1 signaling in hypoxic environments and promotes BCSC-associated phenotypes [66]. Increased levels of the factors such as vascular endothelial growth factor (VEGF) and FGF are not only involved in angiogenesis, but also support the self-renewal and survival of CSCs [67, 68]. The “abnormalization” of angiogenesis that results from dysregulation of these angiogenic factors, which exacerbate hypoxia, is discussed in detail in the section of “Angiogenesis”. Characteristics of CSC and the formation of CSCs under hypoxic conditions are shown in Fig. 3.

**Table 1:** The mechanisms associated with the development of radioresistance in CSCs under hypoxia

CSC-based mechanisms	Cancer type	References
Increasing DNA dependent protein kinase (DNA-PK) activity.	Laryngeal squamous carcinoma	[65]
	Cervical cancer	[69]
Activation of the checkpoint response and improvement to DNA repair	Cervical cancer	[70]
Up-regulated the Twist1, nuclear EGFR localization	Cervical cancer	[69]
Activation of IGF1Rβ/PI3K/Akt pathway	Non-small cell lung cancer	[71]
Increasing autophagic activity	Breast cancer	[72]
Enhancing the expression of HIF-1α	Head and neck squamous cell carcinoma	[73]
	Hepatocellular carcinoma	[74]
Increased expression of HIF-2α mRNA and miR-210	Glioma	[75]
Down-regulated miR-18a-5p	Lung cancer	[76]
Activation of NF-κB/HIF-1 signaling pathway	Laryngeal squamous cell carcinoma	[77]
Over expression of LncRNA PCGEM1	Gastric cancer	[78]
Cause immune escape	Triple negative breast cancer	[79]
Activation of the PI3K/AKT/mTOR signaling	Hepatocellular carcinoma	[21]

CSCs can undergo OXPHOS or glycolysis depending on the cellular state and microenvironment, and can be quiescent by maintaining minimal energy expenditure, thereby enhancing resistance to chemotherapy or radiotherapy [80]. Plasticity of energy metabolism regulates the balance between gain and loss of stemness in tumor cells [81]. Besides, and the CSCs of various tumors are glycolysis-dependent [82–84]. CSCs require glycolysis and lipid metabolism for energy and preferentially use glycolysis to maintain homeostasis [85, 86]. It has been shown that radioresistant medulloblastoma stem-like clones (rMSLCs) have a higher rate of conversion from pyruvate to lactate and a lower rate of conversion from pyruvate to acetyl CoA. Dichloroacetate (DCA) treatment inhibits the glycolysis of rMSLCs and increases radiosensitivity [87]. Pigenin attenuates glycolysis by inhibiting HIF-1 expression, thereby increasing the radiosensitivity of glioma stem cells [88]. Similarly, targeting the ALDH1A3-mediated glycolytic pathway in glioma stem cells improves the outcome of radiotherapy [89]. In addition to the above examples directly related to radioresistance, energy metabolic reprogramming is also able to participate in the formation and maintenance of CSCs, and the following evidence can also provide additional references for the relationship between energy metabolic reprogramming, CSCs and radioresistance. Tumor necrosis factor receptor associated protein 1 (TRAP1), a member of the HSP90 subfamily, is able to regulate the stemness of colorectal carcinoma cells through the Wnt/β-catenin pathway [90]. It also increases aerobic glycolysis and inhibit mitochondrial respiration; the opposite result is produced when TRAP1 is absent [91]. Aerobic glycolysis in cancer cells also enhances the secretion of exosomes [92], which is conducive to the formation of CSCs. Recent studies showed that the PI3K/AKT signaling axis can increase glycolysis and lactate production [93], and upregulate the number of CSCs in nasopharyngeal carcinoma [94]. PI3K/AKT signaling axis may be an important pathway linking CSCs and reprogramming of energy metabolism during malignant development of tumor cells.

During long-term or batch continuous irradiation, some CSC-like cells migrate and infiltrate into the surrounding blood vessels under hypoxia, and are transported to other regions through the blood and lymphatic system [95–97]. The migration of CSC-like cells from primary tumors during radiotherapy is related to poor prognosis and increased risk of metastasis. Photon radiation can induce EMT and increase the proportion of CSC-like cells [62, 98]. On the other hand, proton beam radiation has been shown to reduce the proportion of CSC-like cells and their ability to migrate [99]. This can be attributed to the increased expression of calreticulin on the surface of the irradiated CSCs, which activates the cytotoxic T-lymphocytes against the surviving CSCs. In addition, carbon ion radiotherapy can efficiently eradicate high-grade glioma cells and CSCs, and reduce immune escape under hypoxic conditions [100]. Therefore, proton and carbon ion radiotherapy may provide a better therapeutic strategy for the clearance of CSCs.

### Oxidative stress relief

**Fig. 4:**
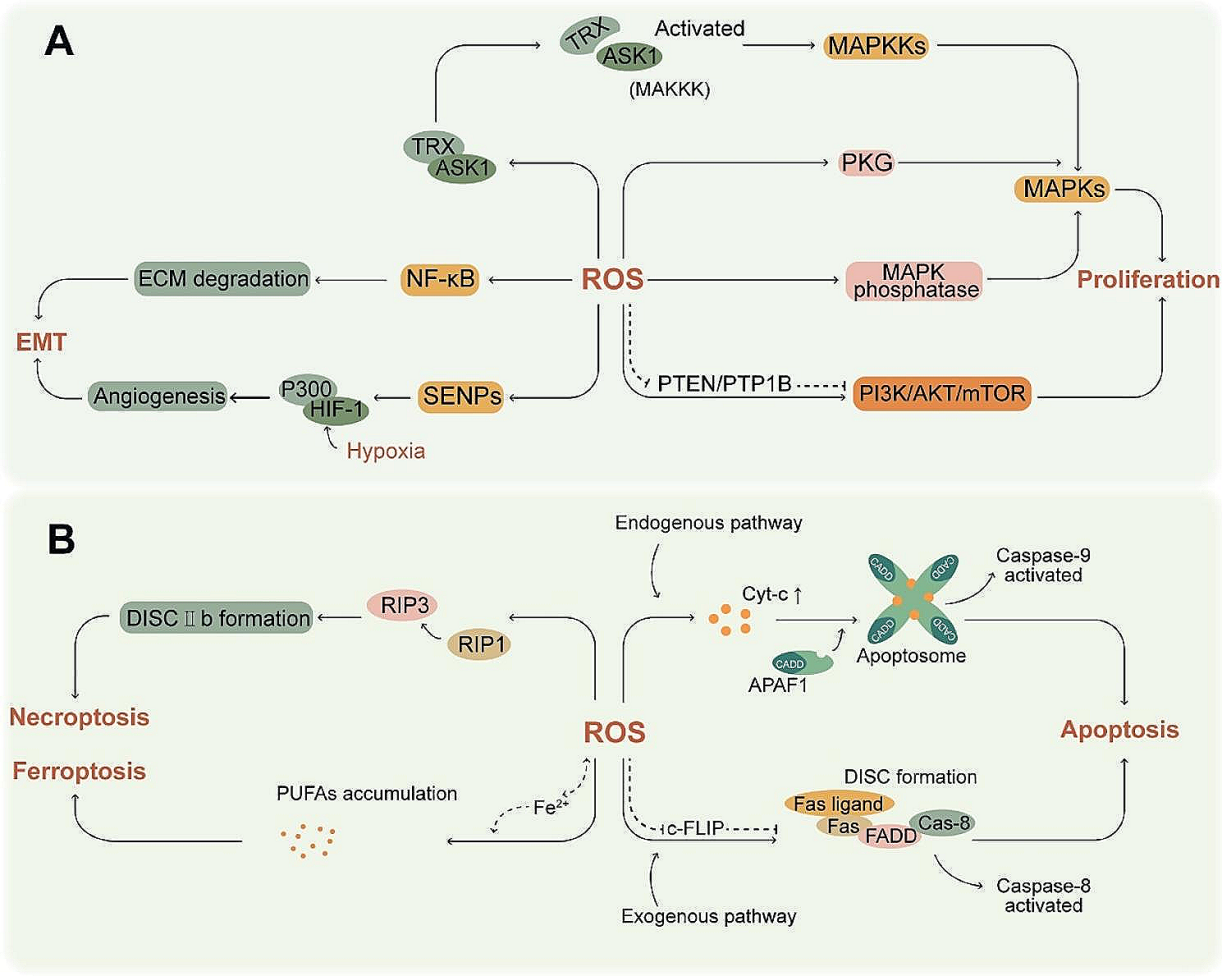
Paradoxical role of ROS in cancer cells. (A) ROS promotes cancer development by activating pathways related to proliferation and EMT. (B) ROS can also inhibit cancer cells by triggering apoptosis through endogenous and exogenous pathways, as well as necroptosis and ferroptosis

Ionizing radiation triggers ROS production and oxidative stress in the tumor cells, resulting in DNA damage [11]. The cellular response to radiotherapy depends on the ability to scavenge ROS and repair DNA damage. As shown in Fig. 4, ROS have the paradoxical role in cancer cells. Therefore, intracellular ROS levels are tightly regulated by the antioxidant system to protect cells from high levels of ROS [101, 102]. Studies show that cells produce higher levels of ROS under hypoxic as opposed to normoxic conditions. For instance, ROS production is increased in GBM8401 and U87 cells cultured under acute hypoxia, which accelerates their growth in vivo and in vitro [103]. Nonetheless, cancer cells can rapidly scavenge ROS under hypoxic conditions due to activation of the antioxidant system, which may contribute to radioresistance. HIF-1 increases GSH levels and enhances radioresistance under hypoxic conditions [22, 104]. GSH is the core endogenous antioxidant that adapts cells to oxidative stress [105]. Piperlongumine (PL) can overcome hypoxia-induce radioresistance of cancer cells by inhibiting thioredoxin and glutathione S-transferase [106], thereby inducing excess ROS production. Buthionine sulfate (BSO) inhibits the rate limiting enzyme glutamate-cysteine ligase involved in GSH synthesis, and has been shown to deplete GSH in the hypoxia tumor areas [107]. Auranofin (AF) is an irreversible inhibitor of thiodoxin reductase, and can attenuate the radioresistance of hypoxic tumors by inducing ROS-mediated DNA damage, mitochondrial dysfunction, and apoptosis. Furthermore, AF has been shown to amplify the radiotherapeutic effects of BSO when used in combination [108], this radiation sensitizing effect is undoubtedly related to the multi-target inhibition of activated antioxidant system induced by hypoxia. There is evidence that ROS can regulate the expression of HIF-1 and VEGF, with important roles in angiogenesis and tumor growth [109].

Given their role in metabolism, the mitochondria are key players in the radioresistance of cancer cells induced by metabolic reprogramming. One study identified 31 differentially expressed mitochondrial proteins in irradiated cancer cells, such as solute carrier family 25 member 22 (SLC25A22) and peroxisomal biogenesis factor 5 (PEX5) [110]. SLC25A22 is able to confer radioresistance to cancer cells by rewiring metabolism [111]. PEX5 can increase radioresistance through activation of the Wnt/β-catenin signaling [112]. Furthermore, cancer cells tolerate hypoxia by accelerating the conversion of ROS, which protects mitochondrial functions [113]. The mitochondrial respiratory chain and active NADPH oxidases (NOXs) are the most prominent endogenous sources of ROS [114, 115]. In the clinical setting, drugs targeting mitochondria have been able to overcome radioresistance of hypoxic tumors. Dichloroacetate (DCA), an inhibitor of mitochondrial pyruvate dehydrogenase kinases (PDHK), can alter tumor metabolism by increasing ROS production in the mitochondria and inhibiting glycolysis [87]. In addition, targeting enzymes in the mitochondrial electron transport chain can also disrupt ROS homeostasis. Arsenic trioxide is an inhibitor of mitochondrial complex IV, which can reduce the level of GSH in radioresistant cancer cells under hypoxia and increase intracellular ROS production, thus reversing radioresistance [116]. However, increasing ROS levels is not a viable strategy for cancer treatment since it will undoubtedly cause systemic toxicity and damage normal cells. Nevertheless, selective disruption of the redox homeostasis in cancer cells by targeting key enzymes involved in metabolic reprogramming may reverse hypoxia-induced radioresistance.

### Autophagy regulation

**Fig. 5:**
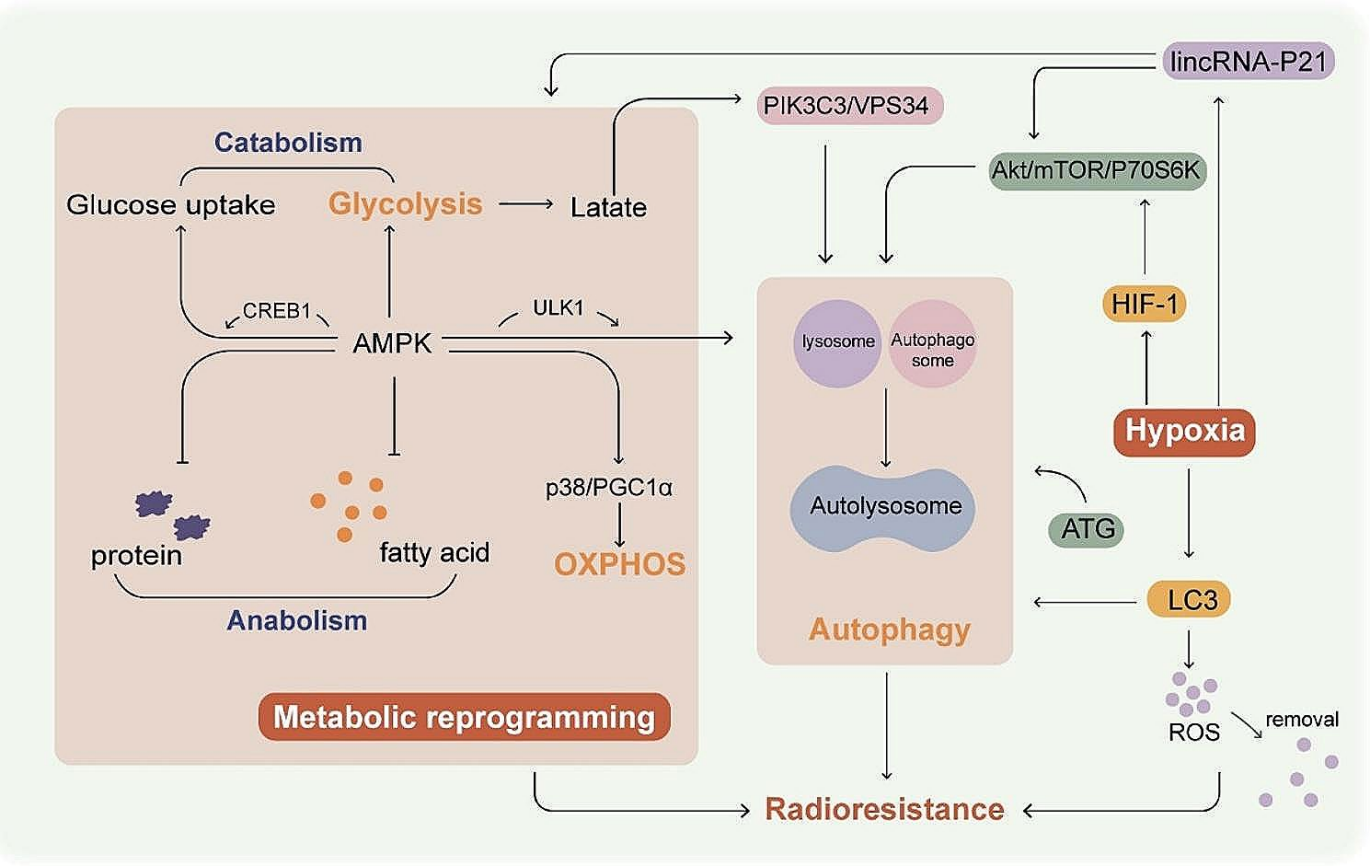
The role of autophagy in radiation therapy under hypoxic conditions and the influence of metabolic reprogramming. In the regulation of energy metabolism, AMPK acts as an energy metabolism “checkpoint”, regulates both OXPHOS and glycolysis, and tightly links energy metabolic reprogramming to autophagy. Moreover, lactate plays a role as a bridge between autophagy and glycolysis. Autophagy is upregulated under hypoxic conditions, exacerbating the effects of radiotherapy

Autophagy is a self-catabolic process wherein cytoplasmic components are engulfed in vesicles, and are degraded following fusion with lysosomes. It is activated in stressed cells, and can either promote cell survival or lead to cell death [117]. It has been shown that autophagy is closely related to the maintenance of pluripotency of CSCs. Inhibition of autophagy greatly reduces pluripotency and promotes CSC differentiation or senescence [118]. In addition, inhibition of autophagy may sensitize tumor cells to radiotherapy under hypoxia stress. One study showed that autophagy-defective head and neck squamous cell carcinoma (HNSCC) cells lacking ATG12 have reduced hypoxia tolerance, and are sensitive to anti-cancer therapies [119]. Under hypoxic conditions, LC3 has been shown to activate autophagy and accelerate the removal of cellular ROS, thereby conferring cells with resistance to irradiation [14]. It has also been shown that in breast cancer cells, hypoxic exposure can elevate autophagic activity and is associated with increased radioresistance [120]. Hypoxia also upregulates autophagy by activating the HIF-1/Akt/mTOR/P70S6K pathway and upregulates radioresistance in cancer cells [121]. In addition to the above examples, other relevant mechanisms of the increased radioresistance mediated by autophagy under hypoxic conditions are summarized and showed in Table 2. The AMPK-ULK1 axis plays an integral role in the activation of autophagy in cancer cells [122]. Furthermore, AMPK also regulates glucose metabolism with cAMP response element binding protein-1(CREB1) [123], and promotes glycolysis in tumor cells [124, 125]. On the other hand, AMPK can also enhance OXPHOS and mitochondrial biosynthesis via the p38/PGC1α pathway [126]. This suggests that AMPK functions as a “checkpoint” for metabolism and regulates both glycolysis and OXPHOS, as well as being an important bridge connecting energy metabolism conversion and autophagy in tumors. It has also been suggested that high lactate mediates PIK3C3/VPS34 emulsification and induces autophagy, thereby promoting cancer progression. Lactate is a bridge linking glycolysis and autophagy [127]. Furthermore, canagliflozin (CAN) can inhibit glucose uptake and lactate release, and modulate autophagy at the same time, thus enhancing the radiosensitivity of HepG2 cells. So, it is considered necessary to perform CAN treatment before radiotherapy [128]. Long intergenic non-coding RNA (lincRNA)-p21 is activated in response to hypoxia, and is a regulator of the cell cycle and Warburg effect. Knockdown of lincRNA-p21 in hepatocellular carcinoma and glioma cells promotes apoptosis, reduces proliferative capacity, and decreases autophagy under hypoxic conditions via the HIF-1/Akt/mTOR/P70S6K pathway [129]. Autophagy, as a cellular self-protective behavior, is able to resist radiation in hypoxic environments through various mechanisms. The acidic environment caused by glycolysis results in an up-regulation of autophagy. Figure 5 shows the autophagy-induced mechanisms causing radioresistance under hypoxic conditions and associated with energy metabolic reprogramming, as well as their interaction pathways.

**Table 2:** The autophagy-based mechanisms of radioprotection in hypoxia-adapted cancer cells

Autophagy-based mechanisms	Cancer type	References
Increasing DNA damage repair	Breast cancer cells	[130]
	Breast cancer cells	[120]
Parkin-mediated digesting mitochondria	Breast cancer cells	[131]
Reducing ROS	Osteosarcoma cells	[14]
	Lung cancer cells	[132]
Activating HIF-1/Akt/mTOR/P70S6K pathway	Hepatoma and glioma cells	[129]
	Breast cancer cells	[121]
Activating HIF-1, c-Jun	Lung cancer cells	[133]
HIF-1α/miR-210/Bcl-2 pathway	Colon cancer cells	[134]
MiR-124 and miR-144 downregulation	Prostate cancer cells	[135]
Higher level of miR-301a and miR-301b expression	Prostate cancer cells	[136]
YAP over-expression promoted the transcription and translocation of HMGB1	Glioblastoma cells	[137]
Activation of the unfolded protein response (UPR)	Colon cancer, breast cancer and glioma cells	[138]
Overexpression of p63	Oral squamous cell carcinoma	[139]
Activating the AMPK/mTOR pathway	Nasopharyngeal carcinoma	[140]

Several additional studies, however, have hinted at the role of autophagy on the opposite side of radioresistance. Cancer cells resistant to apoptosis may use autophagy as a primary response to ionizing radiation. Radiosensitization induced by inhibition of NF-κB is associated with autophagy; conversely, inhibition of autophagy decreases radiosensitization [141]. Consistent with this, inhibition of autophagy increased radioresistance of cervical cancer cells [142] and colorectal cancer cells [143]. Furthermore, hypoxia can enhance autophagy under high cell density and downregulate EGFR, leading to cell death and radiosensitization [144]. The PI3K/mTOR pathway inhibitor NVP-BEZ235 sensitized breast cancer cells to radiotherapy under hypoxic conditions by inducing autophagy [145]. In contrast, other studies have shown that autophagy promotes radioresistance of breast cancer cells [120, 121, 130, 131], and autophagy inhibitors like chloroquine can increase the radiosensitivity of hypoxic cancer cells. Therefore, the role of autophagy in hypoxic tumors may depend on various factors. Nevertheless, autophagy regulation is an essential target for overcoming radioresistance in hypoxic cancer cells and is closely related to metabolic reprogramming.

### Angiogenesis

Tumor growth is accompanied by the continuous generation of new blood vessels to help sustain the rapidly proliferating cancer cells [146], and abnormal, pathological angiogenesis is often associated with tumor invasion and metastasis [147]. It has long been recognized that the tumor vasculature is functionally and structurally heterogeneous, with a haphazard distribution, irregular branching, and the formation of arteriovenous shunts [148]. Traditionally, it was believed that anti-angiogenic agents would inhibit tumor angiogenesis, depriving the tumor of essential nutrients and oxygen. However, studies have shown that in tumor, excessive angiogenic factors can cause poor and disturbed vascular blood flow and leakage, leading to poor drug delivery and hypoxia [149]. In this pathological condition, angiogenic factors, acting as the “abnormalization factor”, promote a vascular “abnormalization switch” [150]. This abnormal and pathologically excessive angiogenesis may also be an important contributor to radioresistance. Anti-angiogenic therapy has been suggested to alter the structural and functional defects of the tumor vasculature, a process known as “vascular normalization” [151]. A previous study has shown that the use of the anti-angiogenic agent SU6668 increases radiosensitivity [152].

Insufficient oxygen supply and the resulting reduction in tissue oxygen tension often lead to angiogenesis to satisfy tissue needs [153]. Hypoxia upregulates the pro-angiogenic VEGF [154], placental growth factor (PlGF) and fibroblast growth factor (FGF) [155]. HIF-1α complexes with other molecules such as HIF-1β to enhance erythropoietin transcription [156]. So, hypoxia is a central driver of angiogenesis [157]. The angiopoietin (Ang)-1 maintains vascular integrity, and inhibits Ang-2 expression in normal adult tissues. In the presence of VEGF and HIF-1, Ang-2 acts as an antagonist of Ang-1, disrupting the normal balance of angiogenesis, and increased proliferation and migration of endothelial cells (ECs) leads to vascular instability and pathological angiogenesis [158–160]. Based on the above mechanisms of abnormal angiogenesis under hypoxia, many studies have demonstrated the radiosensitizing effect of treatments targeting angiogenesis-related factors. Fucoidan-coated manganese dioxide nanoparticles (Fuco-MnO_2_-NPs) are able to inhibit the expression of phosphorylated vascular endothelial growth factor receptor 2 (VEGFR2) and CD31, overcoming radioresistance through dual targeting of tumor hypoxia and angiogenesis [161]. Latent membrane protein 1 (LMP1) can increase the expression of VEGF through the JNKs/c-Jun signaling pathway, and LMP1-targeted DNAzyme (DZ1) can enhance the radiosensitivity of nasopharyngeal carcinoma (NPC) cells by inhibiting the activity of HIF-1/VEGF [162]. Besides, in a preclinical study, Motesanib (a potent inhibitor of VEGFR-1, 2, and 3, PDGFR, and Kit receptors) significantly improves intertumoral hypoxia and achieves better therapeutic results when combined with radiation [163]. In addition, interstitial fluid pressure (IFP) is elevated in solid tumors, and angiogenesis inhibitors can reduce IFP to morphologically and functionally “normalize” the vascular network, overcoming hypoxia, generating more free radicals, leading to more DNA damage, and increasing the sensitivity to radiotherapy [164].

Tumor ECs have highly glycolytic metabolism. Inhibition of glycolysis activator PFKFB3 in endothelial cells induces normalization of tumor vasculature, inhibits metastasis and improves therapeutic outcome [165]. Pericytes help stabilize the vascular structure and support ECs through gap junctions [166, 167]. It has been shown that hexokinase 2 (HK2)-driven glycolysis is elevated in tumor pericytes, which upregulates their ROCK2-MLC2-mediated contractility, leading to impaired vascular support function [168]. Lactate dehydrogenase (LDH-5) catalyses the conversion of pyruvate to lactate. Studies have shown that LDH-5 is highly expressed in endometrial adenocarcinomas and is strongly associated with the expression of phosphorylated VEGFR2/KDR receptors in tumour-associated blood vessels. Administration of VEGF- tyrosine kinase receptor inhibitors may be an adjuvant to radiotherapy and chemotherapy [169]. In terms of energy metabolic reprogramming, glycolysis exacerbates vascular abnormalities, which results in even more intensified hypoxia within tumor.

**Fig. 6:**
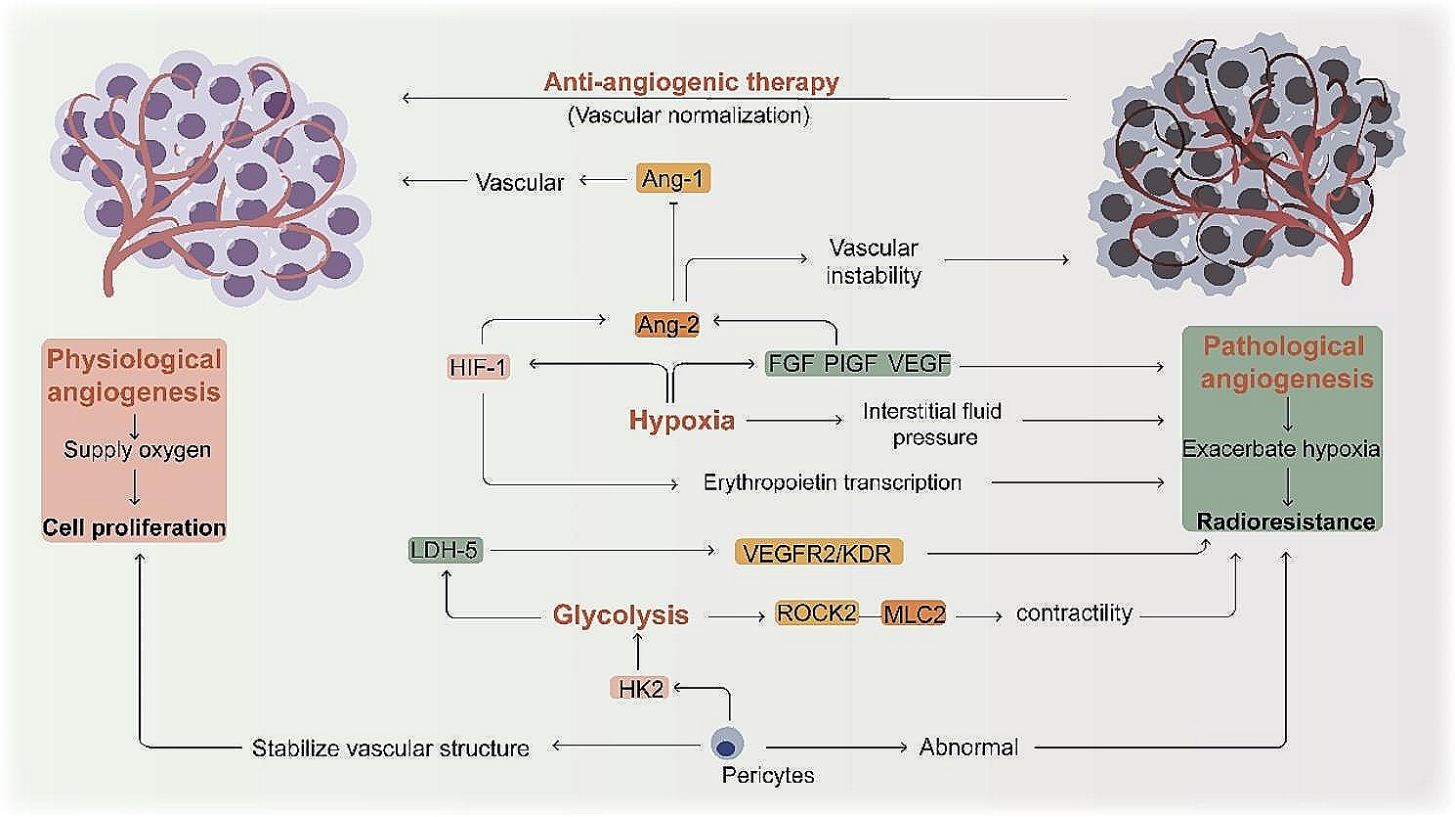
Physiological and pathological angiogenesis in tumors. Under hypoxic conditions, angiogenic factors such as VEGF are upregulated, resulting in dysregulation of Ang-2 and Ang-1, leading to excessive angiogenesis and abnormalisation. Glycolysis affects pericyte function as well as the expression of LDH-5 in cancer cells, thus exacerbating the abnormal vascularisation. This pathological angiogenesis exacerbates hypoxia and contributes to the development of radioresistance. Antiangiogenic therapy can reverse this process and in combination with radiotherapy can have a radiosensitizing effect

Angiogenesis under the influence of hypoxia and reprogramming of energy metabolism, regulated by angiogenic factors, is shown in Fig. 6. Both pre-existing normal blood vessels and neovascularisation may support tumor growth and progression. In contrast, excessive angiogenesis vascular “abnormalization” under pathological conditions can exacerbate hypoxia and contribute to radioresistance. Elucidation of the molecular mechanisms of pathological angiogenesis and the homeostatic regulation of angiogenic factors may provide new targets for improving the radiosensitivity of cancer cells. Tumor growth and angiogenesis are an interdependent cycle that can be broken by antiangiogenic therapy, thereby reducing radioresistance [170]. However, irradiation and anti-angiogenic therapies can cause angiogenesis to switch from sprouting to intussusception. This is a protective response of the tumour and is responsible for the development of resistance and rapid recovery after cessation of treatment [171]. For anti-angiogenic therapy, how to adjust the optimal dose and course of treatment in order to normalise the tumour vasculature without harming normal tissues and how to be able to achieve radiosensitiszation are questions worth pondering.

### Immune escape

Tumor growth is largely dependent on the inability of the immune system to eliminate the malignant cells. Radiotherapy can affect the TME, which includes the immune system and associated cells [172]. Irradiation promotes the formation of an anti-immunogenic microenvironment by recruiting tumor-associated macrophages (TAMs) and myeloid-derived suppressor cells (MDSCs) [173–175]. In additional, there is the involvement of Tregs [176], DCs [177], and some molecules such as transforming growth factor-β (TGF-β) [178], and C-C motif chemokine ligand 2 (CCL2) [179]. Programmed death-ligand 1 (PD-L1) is expressed on cancer cells and binds to programmed cell death-1 (PD-1) on immune cells, resulting in an immunosuppressive signal that inhibits lymphocyte activation. The PD-1/PD-L1 checkpoint limits the immune response against multiple cancer cells [180]. PD-L1 upregulation prevents activation of T cells and NK cells [181]. Therefore, PD-L1-mediated immune escape is also an important cause of radioresistance [182–184]. Mechanisms underlying immunosuppression caused by radiation are summarized in Table 3.

**Table 3:** Mechanisms underlying immunosuppression caused by radiation

Immune escape mechanism	Cancer type	References
Programmed cell death ligand 1(PD-L1) activation	Melanoma	[182]
	Cutaneous squamous-cell carcinoma of the head and neck area (cSCC-HN)	[183]
	Prostate cancer	[184]
CircIGF2BP3 reduces PD-L1 ubiquitination	Non-small cell lung cancer	[185]
DNA repair mitigates radiation-induced replication stress	Breast cancer	[186]
Effect of regulation factor TGF- β, Functions of MIF, CCL2, CXCL5, CXCL8 and CXCL12	Rhabdomyosarcoma	[187]
STAT3 serine 727 phosphorylation	Glioblastoma	[188]
CSC causes NK cells lose cytotoxicity	Triple negative breast cancer	[79]
Upregulation of B7-H3 on circulating epithelial tumor cells (CETCs)	Breast cancer	[189]
Lactate regulates dendritic cell activation	Melanoma and prostate carcinoma	[190]
Host STING-dependent MDSC mobilization	Colon cancer	[191]
Activation of noncanonical NFκB pathway through the cGAS-STING DNA	Colon cancer	[192]
Activation of TGFβ	Breast cancer	[178]
Up-regulation of Treg cells	Prostate cancer	[176]
Regulation of the Treg-dendritic cell axis	Head and neck squamous cell carcinoma (HNSCC)	[177]
Effect of Mac-1 (CD11b/CD18)	Squamous cell carcinoma	[173]
MDSCs impair the activity of T cells	Lung cancer	[193]
Generation of CCL2	Pancreatic ductal adenocarcinoma	[179]
M2 differentiated tumor macrophages	Breast cancer	[175]
	Prostate cancer	[174]

Under hypoxic conditions, HIF-1α upregulates PD-L1 on cancer cells and MDSCs, thereby interfering with T cell effector function [194]. Much evidence suggests that hypoxic environments also have a regulatory effect on immunosuppressive cells. It has also been found that terminally depleted CD8^+^ T cells and immunosuppressive cells, including Treg cells and M2 TAMs, are enriched in the core region of hypoxia to a greater extent than in the peripheral region [195]. Hypoxic tumor promotes the recruitment of Tregs via CCL28, which in turn suppresses the function of effector T cells [196]. Hypoxia significantly alters MDSC function in the TME via HIF-1α and differentiation towards TAMs [197]. Moreover, TAMs inhibit T cell function under hypoxic conditions in a HIF-1α-dependent manner [198]. Hypoxia also promotes the development of M2 macrophages, resulting in immunosuppression and decreased radiosensitivity [174]. Signal transducer and activator of transcription 3 (STAT3) can be activated by HIF-1 under hypoxic conditions [199]. Sustained activation of the STAT3 signaling promotes cell proliferation, metastasis and immune escape. STAT3 inhibitors suppress STAT3 activation, down-regulate HIF-1α expression, and up-regulate the radiosensitivity of esophageal squamous cell carcinoma (ESCC) in vivo and in vitro [200]. Besides, HIF-1α is a metabolic switch between glycolysis-driven migration and oxidative phosphorylation-driven immunosuppressive colonization in glioblastoma [201]. In summary, hypoxic conditions are more favorable for immune escape to occur.

Metabolic reprogramming plays a central role in the immune escape of tumor cells [202]. The TME consists of stroma and various components of the immune system, and alterations in the microenvironment that lead to metabolic reprogramming inhibit immune cell activity against cancer cells [203]. The activated T cells also produce ATP through aerobic glycolysis following induction of LDHA, in order to reduce the burden on mitochondria [204]. In T effector cells, LDHA is induced via PI3K-Akt-Foxo1 signaling, which in turn is regulated by glycolytic ATP [205]. Furthermore, LDHA controls the immune microenvironment by regulating the function of MDSCs [206]. Tumor-derived d-2-hydroxyglutarate (d-2HG) is taken up by CD8^+^ T cells, resulting in metabolic changes and a decrease in immune function via LDH [207]. CD8^+^ T cell depletion can also be induced by mitochondrial dysfunction produced by prolonged hypoxic stimulation [208]. Lactate promotes immune escape by inhibiting migration of monocytes, the precursors of TAMs, and the secretion of tumor necrosis factor (TNF) and interleukin-6 (IL-6) [209, 210]. Lactate can also inhibit the function of T cells and NK cells [211]. Furthermore, the upregulation of glucose transporter 1 (Glut1) in Treg cells via TLR (Toll-like receptor) increases glucose uptake and lactate production, which suppresses the effector T cells and DCs, thereby enhancing cancer cell survival [212]. However, Treg cells are not affected by pH value and lactate levels [213]. These evidences suggest that the ability of factors involved in reprogramming energy metabolism and the acidic environment to influence the ability of immune cells, thus preventing complete clearance of cancer cells.

**Fig. 7:**
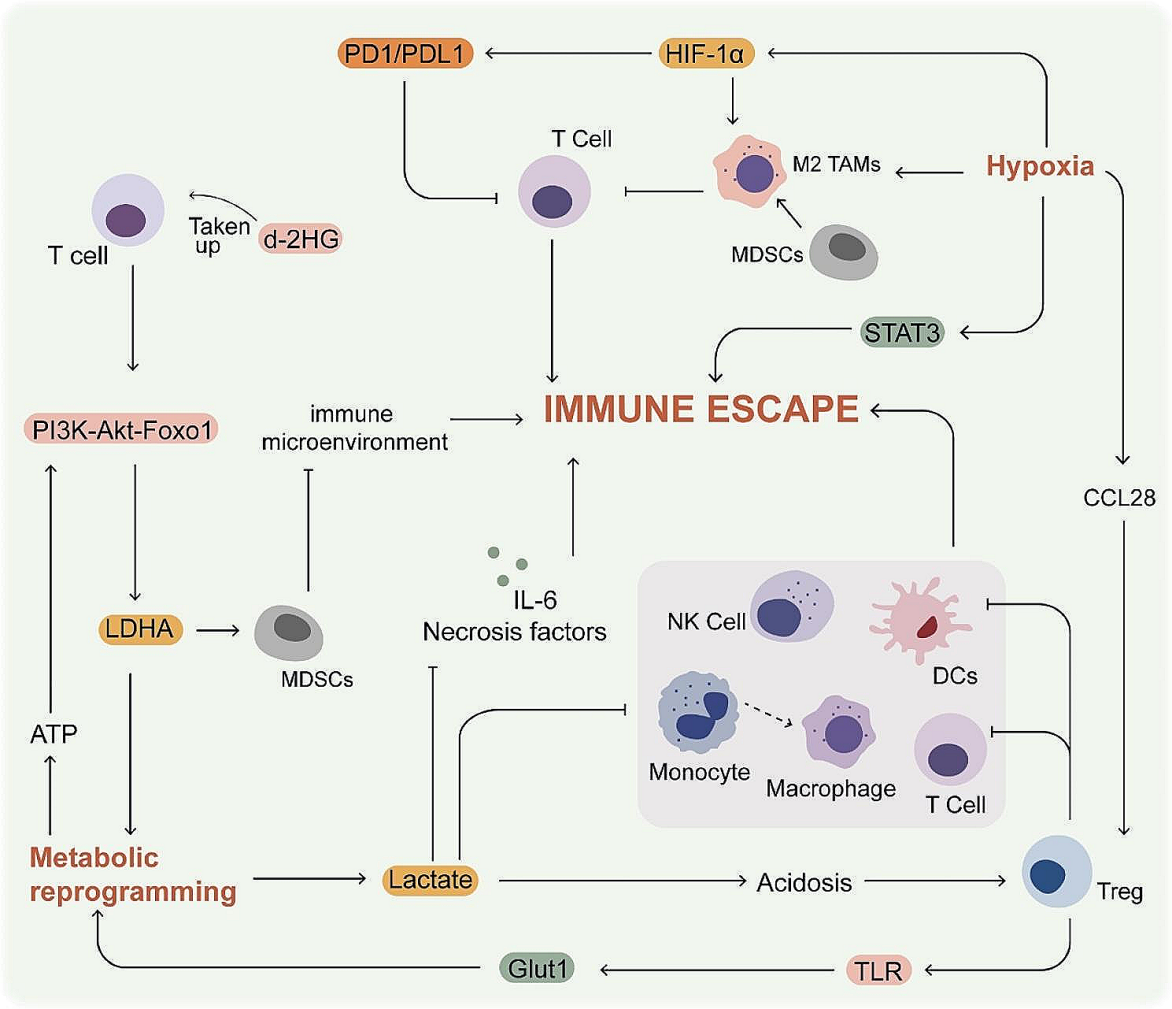
Relationship between hypoxia, metabolic reprogramming and immune escape of cancer cells. The PD1/PDL1 axis is an immune checkpoint that is activated by HIF-1α in hypoxic TME. Hypoxia also inhibits T-cell function by promoting differentiation of M2 TAMs and activation of STAT3, thereby promoting immune escape. Lactate inhibits immune cell function and thus promotes immune escape. MDSCs are affected by LDHA, thus suppressing the immune microenvironment. Treg cells can be up-regulated by hypoxia and lactate, and exert immunosuppressive effects. Treg cells also increase Glut1 levels by up-regulating TLR, thus promoting glucose uptake and lactate production

The relationship between hypoxia, metabolic reprogramming and immune escape of cancer cells is depicted in Fig. 7. To summarize, the acidic and hypoxic TME modulates immunosuppressive cells and affects the functioning of immune cells to perform their functions, thus allowing cancer cells to evade the immune system and probably even leading to the development of radioresistance. Therefore, the combination of radiotherapy and immunotherapy is a promising strategy for cancer treatment.

### The feedback loop between metabolic reprogramming and hypoxia

**Fig. 8:**
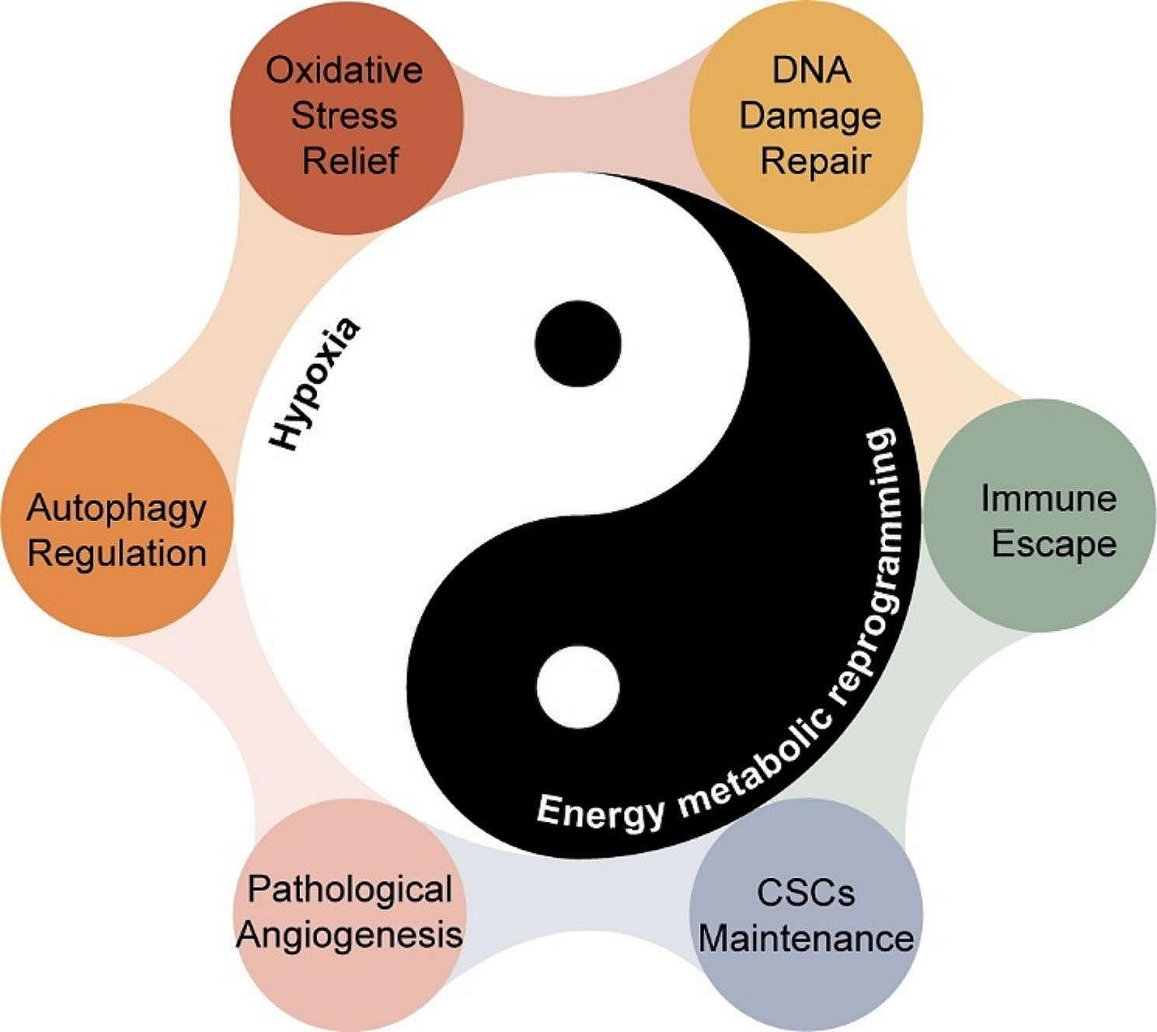
“Taiji diagram” showing the relationship between metabolism reprogramming and hypoxia. Black and white represent “yin” (energy metabolic reprogramming) and “yang” (hypoxia) respectively. Metabolic reprogramming and hypoxia form a synergistic relationship regulated by oxidative stress, angiogenesis, CSCs maintenance, immune escape and DNA repair to promote radioresistance

Studies increasingly show that cancer is a metabolic disorder. During tumor development, genetic mutations lead to metabolic reprogramming that efficiently produces ATP, macromolecules and organelles, and activates autophagy to sustain the high proliferation rates. In addition, the Warburg effect is also associated with the activation of the antioxidant system and pathological angiogenesis, resulting in hypoxia. The hypoxic environment induces HIF-1, exosomes and HSPs, which further increases metabolic reprogramming and glycolysis in cancer cells. As shown in Fig. 8, This feedback loop between metabolic reprogramming and hypoxia is akin to “yin” and “yang” in the traditional Chinese “Taiji diagram”, which drives radioresistance and the malignant progression of tumors.

**Fig. 9:**
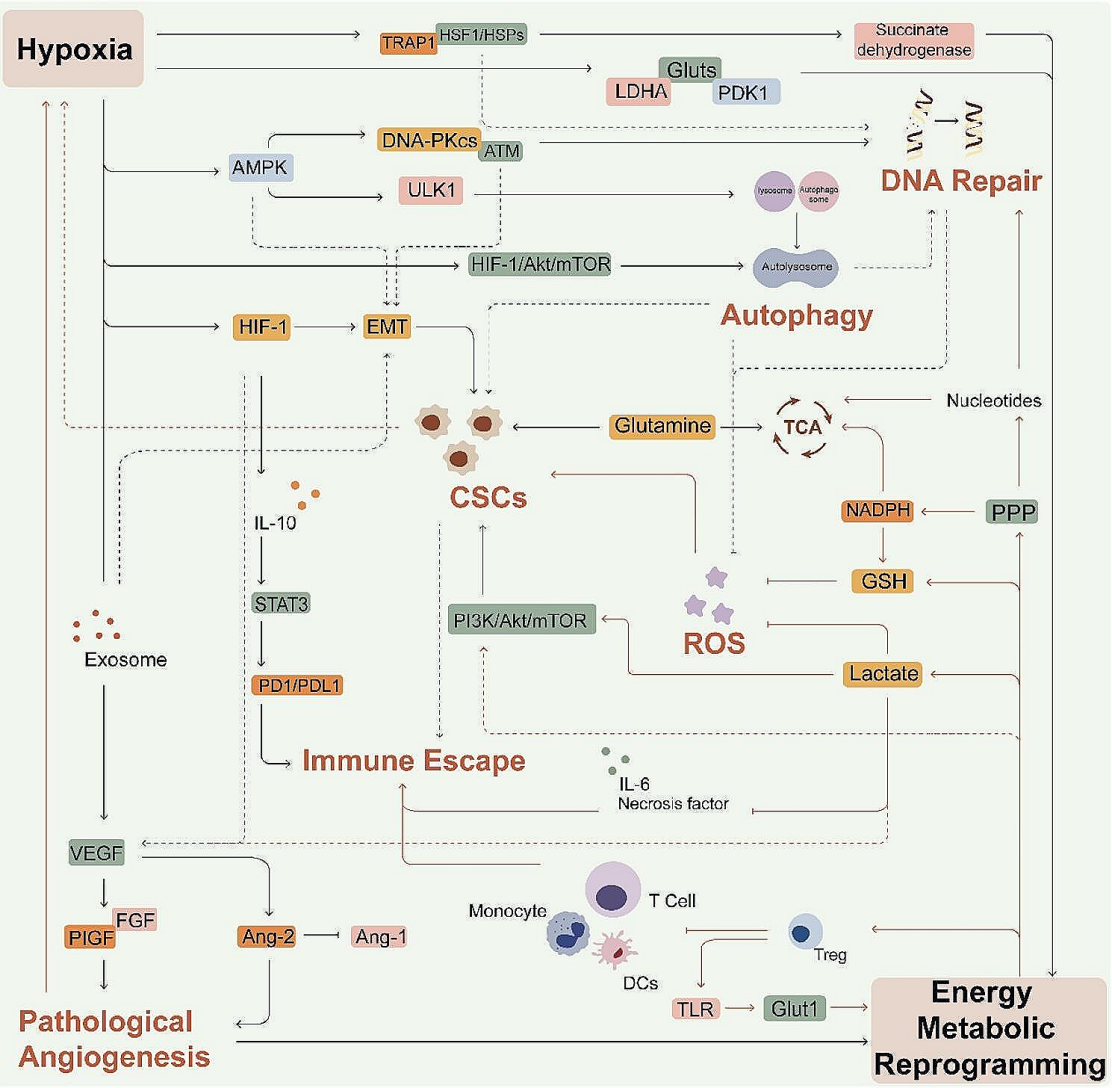
The feedback loop between energy metabolic reprogramming and hypoxia. The key molecules, metabolites, and signaling pathways linking hypoxia and glycolysis are illustrated. This vicious cycle of hypoxia and metabolic reprogramming makes the tumor cells recalcitrant to radiotherapy. The orange line indicates the direction of metabolic reprogramming → hypoxia, and the black line indicates the direction of hypoxia → metabolic reprogramming. The dotted lines indicate more distant relationships

The detailed feedback loop is shown in Fig. 9. Under hypoxic conditions, HIF-1 increases glucose uptake and reduces metabolite entry into the TCA cycle by upregulating GLUTs, pyruvate dehydrogenase kinase 1 (PDK1) and LDHA. This inhibits mitochondrial respiration and reduces acetyl Co-A production, increases glycolysis and lactate levels [214], and activates the antioxidant system. A previous study identified a positive feedback loop consisting of p21/HIF-1α that exacerbates radioresistance in glioblastoma cells by promoting Glut1/LDHA-mediated glycolysis [215]. In addition, HIF-1 induces HSP and activates the synthesis of ribonucleotides, thus increasing DNA repair and promoting radioresistance. The feedback loop between metabolic reprogramming and hypoxia also activates the HIF-1, AMPK and PI3K\AKT\mTOR pathways, and maintains CSCs by activating autophagy and EMT, which eventually attenuate radiosensitivity as described in the preceding sections. In addition, the Warburg effect results in lactate accumulation that induces VEGF production by ECs, resulting in the formation of hyperplastic vessels that cannot supply sufficient oxygen to the rapidly proliferating cells, and thus aggravate hypoxia. Lactate overload also suppresses immune cell activity, which along with hypoxia-induced PD-L1 expression, aids in the immune escape of cancer cells. Therefore, hypoxia and metabolic reprogramming synergistically enhance the radioresistance of cancer cells and promote tumor progression.

## Discussion and prospects

Metabolic reprogramming and hypoxia are the hallmarks of tumor initiation and progression. Cancer cells switch to glycolysis, which is independent of oxygen supply and the mitochondria, as the main source of energy to sustain their high proliferation rates since it is a simpler process compared to mitochondrial OXPHOS. Hypoxia and metabolic reprogramming form a complex, multidirectional loop that can induce radioresistance through DNA repair, autophagy, maintenance of CSCs, immune escape, angiogenesis, and oxidative stress relief. HIF-1 and lactate regulate almost all mechanisms that generate radioresistance. Some of the pathways involved in this loop counteract the effects of radiation therapy by supporting cell proliferation and blocking apoptosis, while the others protect cancer cells from radiation damage by decreasing ROS production or reducing oxygen supply through pathological angiogenesis.

Although tumor cells can be sensitized to radiation by targeting specific pathways, there is currently no drug that can effectively reverse radioresistance. Since most studies have focused on the effects rather than the regulators of the feedback loop between metabolic reprogramming and hypoxia, the clinical consequences of blocking a specific pathway are not completely clear. For example, while HIF-1 inhibitors may improve the outcomes of radiotherapy, severe and prolonged dysfunction of HIF-1 exacerbates tumor hypoxia by full blockade of angiogenesis [9, 216, 217]. Therefore, combining HIF-1 inhibitor with artificial oxygenation is a viable strategy to sensitize hypoxic tumors to radiotherapy [9]. Furthermore, one research group was able to achieve radiosensitization of lung cancer EDB-1 cells and breast cancer MDA-MB-231 cells using nano oxygen bubbles [218]. SLC3A2 is a member of the solute carrier family of proteins, and is expressed in proliferative cells. SLC3A2-deficient HNSCC cells exhibit higher radiosensitivity and increased levels of autophagy, and inhibiting autophagy in these cells through ATG5 knockdown or bafilomycin A1 treatment further increased radiosensitivity. Thus, autophagy inhibition combined with SLC3A2-targeted therapy could be a promising strategy for the radiosensitization of HNSCC cells [219]. Likewise, prostate cancer cells can be sensitized to radiation by inducing glutamine deprivation, which can lead to oxidative stress, DNA damage, depletion of CSCs, and autophagy. Therefore, simultaneous inhibition of glutamine metabolism and autophagy could be a more effective therapeutic strategy [220]. Several targets of the feedback loop have a two-sided role in cancer, and the current research on them is ambiguous. For example, autophagy may cause cell death or facilitate cell survival, and the effect of tumor vasculature depends on whether angiogenesis is physiological or pathological. Nevertheless, the mechanisms underlying these paradoxical effects have to be elucidated in order to regulate the feedback loop between metabolic reprogramming and hypoxia, and reverse radioresistance of cancer cells.

Precision radiotherapy refers to the individualized treatment of cancer patients based on biomarkers and advanced radiotherapy techniques in order to improve treatment outcomes and reduce adverse effects [221]. Based on the studies so far, we can surmise that the feedback loop between hypoxia and reprogramming of energy metabolism might be the root cause of radioresistance. There are no marketed HIF-1 inhibitors for use as anticancer therapy in clinical practice. However, VEGF is a downstream gene of HIF-1, and bevacizumab, which targets VEGF, is already in clinical use. Combination of Bevacizumab and radiotherapy improves overall survival (OS) and reduces radiation necrosis (RN) [222]. The therapeutic effect of bevacizumab is most likely related to overcoming tumor hypoxia and inhibiting excessive angiogenesis. In terms of energy metabolic reprogramming, there are no clinically proven marketed drugs targeting aerobic glycolysis. As early as 1958, researchers showed that 2-deoxy-D-glucose (2-DG), which inhibits glycolysis, had significant adverse side effects and limited efficacy in humans [223]. However, clinical trials of the anti-tumor effects of 2-DG remain promising (NCT00096707, NCT05314933) [224], and significant radiosensitization of 2-DG has been demonstrated in combination with radiotherapy [225, 226]. The use of lactate by cancer cells is dependent on the expression of monocarboxylic acid transporters (MCTs). Clinical trials of the MCT inhibitor AZD3965 in the treatment of B-cell lymphoma cancer are also underway (NCT01791595) [227]. And the combination of AZD3965 with radiotherapy prolongs survival and improves radiosensitivity [228]. These promising drugs combined with radiation may have a significant enhancing effect on the efficacy of radiotherapy in the clinic. However, cancer cells share several metabolic networks with normal cells, and mechanisms that maintain critical metabolic fluxes in cancer cells are currently unknown. How to avoid off-target effects and systemic toxicity is an important issue. Moreover, the significant heterogeneity and complexity of the tumor microenvironment within tumor makes it challenging to identify specific therapeutic targets. A greater understanding of this feedback loop will unearth potential targets for improving radiosensitivity of cancer cells and inhibiting tumor development.

## Data Availability

No datasets were generated or analysed during the current study.

## Abbreviations

CSCs: Cancer stem cells
ROS: Reactive oxygen species
PDH: Pyruvate dehydrogenase
TCA: Tricarboxylic acid cycle
OXPHOS: Oxidative phosphorylation
LDH: Lactate dehydrogenase
HIF-1: Hypoxia-inducible factor-1
GSH: Glutathione
SSB: Single-strand break
DSB: Double strand break
PL: Piperlongumine
BSO: Buthionine sulfate
AF: Auranofin
DCA: Dichloroacetate
PDHK: Pyruvate dehydrogenase kinases
HNSCC: Head and neck square cell carcinoma
AMPK: AMP-activated protein kinase
CREB1: CAMP response element binding protein-1
LincRNA: Long intergenic non-coding RNA
CAN: Canagliflozin
PDAC: Pancreatic ductal adenocarcinoma
UPR: Unfolded protein response
EMT: Epithelial-mesenchymal transition
TRAP1: Tumor necrosis factor receptor associated protein 1
GLS: Glutaminase
ALDH: Aldehyde dehydrogenase
DNA-PK: DNA dependent protein kinase
ECs: Endothelial cells
VEGF: Vascular endothelial growth factor
PlGF: Placental growth factor
FGF: Fibroblast growth factor
DDR: DNA damage response
HBP: Hexosamine biosynthetic pathway
PPP: Pentose phosphate pathway
MUC1: Mucin1
ATM: Ataxia telangiectasia-mutated
DNA-PKcs: DNA dependent protein kinase catalytic subunit
HSF1: Heat shock transcription factor 1
HSPs: Heat shock proteins
HR: Homologous recombination
BCSCs: Breast cancer stem cells
TLS: Translesion synthesis
PD-L1: Programmed death-ligand 1
PD-1: Programmed cell death-1
SPOP: Speckle-type POZ protein
LDHA: Lactate dehydrogenase A
MDSCs: Myeloid-derived suppressor cells
IL-6: Interleukin-6
DCs: Dendritic cells
Glut1: Glucose transporter 1
TLR: Toll-like receptor
cSCC-HN: Cutaneous squamous-cell carcinoma of the head and neck area
CETCs: Circulating epithelial tumor cells
PDK1: Phosphoinositide-dependent protein kinase-1

## References

[1] Shaked Y (2019). The pro-tumorigenic host response to cancer therapies. Nat Rev Cancer.

[2] Herrera FG, Bourhis J, Coukos G (2017). Radiotherapy combination opportunities leveraging immunity for the next oncology practice. CA Cancer J Clin.

[3] Ozpiskin OM, Zhang L, Li JJ (2019). Immune targets in the tumor microenvironment treated by radiotherapy. Theranostics.

[4] Li Y, Sun C, Tan Y, Zhang H, Li Y, Zou H (2021). ITGB1 enhances the Radioresistance of human non-small cell Lung Cancer cells by modulating the DNA damage response and YAP1-induced epithelial-mesenchymal transition. Int J Biol Sci.

[5] Bai X, Ni J, Beretov J, Wang S, Dong X, Graham P (2021). THOC2 and THOC5 regulate stemness and Radioresistance in Triple-negative breast Cancer. Adv Sci (Weinh).

[6] Kutilin D (2021). Genetic and epigenetic bases of prostate tumor cell radioresistance. Klin Onkol.

[7] Peitzsch C, Cojoc M, Hein L, Kurth I, Mäbert K, Trautmann F (2016). An epigenetic reprogramming strategy to Resensitize Radioresistant prostate Cancer cells. Cancer Res.

[8] Viallard C, Larrivée B (2017). Tumor angiogenesis and vascular normalization: alternative therapeutic targets. Angiogenesis.

[9] Kabakov AE, Yakimova AO. Hypoxia-Induced Cancer cell responses driving Radioresistance of Hypoxic tumors: approaches to Targeting and Radiosensitizing. Cancers (Basel). 2021;13(5). 10.3390/cancers13051102 PMC7961562 33806538

[10] Dewhirst MW, Cao Y, Moeller B (2008). Cycling hypoxia and free radicals regulate angiogenesis and radiotherapy response. Nat Rev Cancer.

[11] Gray LH, Conger AD, Ebert M, Hornsey S, Scott OC (1953). The concentration of oxygen dissolved in tissues at the time of irradiation as a factor in radiotherapy. Br J Radiol.

[12] Howard-Flanders P, Moore D (1958). The time interval after pulsed irradiation within which injury to bacteria can be modified by dissolved oxygen. I. A search for an effect of oxygen 0.02 second after pulsed irradiation. Radiat Res.

[13] Zhong J, Rajaram N, Brizel DM, Frees AE, Ramanujam N, Batinic-Haberle I (2013). Radiation induces aerobic glycolysis through reactive oxygen species. Radiother Oncol.

[14] Feng H, Wang J, Chen W, Shan B, Guo Y, Xu J (2016). Hypoxia-induced autophagy as an additional mechanism in human osteosarcoma radioresistance. J Bone Oncol.

[15] Vander Heiden MG, Cantley LC, Thompson CB (2009). Understanding the Warburg effect: the metabolic requirements of cell proliferation. Science.

[16] Finley LWS. What is cancer metabolism? Cell. 2023. 10.1016/j.cell.2023.01.038 PMC10106389 36858045

[17] Warburg O (1956). On the origin of cancer cells. Science.

[18] Shi Y, Wang Y, Jiang H, Sun X, Xu H, Wei X (2021). Mitochondrial dysfunction induces radioresistance in colorectal cancer by activating [Ca(2+)](m)-PDP1-PDH-histone acetylation retrograde signaling. Cell Death Dis.

[19] Kim JW, Tchernyshyov I, Semenza GL, Dang CV (2006). HIF-1-mediated expression of pyruvate dehydrogenase kinase: a metabolic switch required for cellular adaptation to hypoxia. Cell Metab.

[20] Zhang W, Li L, Guo E, Zhou H, Ming J, Sun L (2022). Inhibition of PDK1 enhances radiosensitivity and reverses epithelial-mesenchymal transition in nasopharyngeal carcinoma. Head Neck.

[21] Bamodu OA, Chang HL, Ong JR, Lee WH, Yeh CT, Tsai JT. Elevated PDK1 expression drives PI3K/AKT/MTOR signaling promotes Radiation-resistant and dedifferentiated phenotype of Hepatocellular Carcinoma. Cells. 2020;9(3). 10.3390/cells9030746 PMC7140693 32197467

[22] Nakashima R, Goto Y, Koyasu S, Kobayashi M, Morinibu A, Yoshimura M (2017). UCHL1-HIF-1 axis-mediated antioxidant property of cancer cells as a therapeutic target for radiosensitization. Sci Rep.

[23] Guillaumond F, Leca J, Olivares O, Lavaut MN, Vidal N, Berthezène P (2013). Strengthened glycolysis under hypoxia supports tumor symbiosis and hexosamine biosynthesis in pancreatic adenocarcinoma. Proc Natl Acad Sci U S A.

[24] Busk M, Walenta S, Mueller-Klieser W, Steiniche T, Jakobsen S, Horsman MR (2011). Inhibition of tumor lactate oxidation: consequences for the tumor microenvironment. Radiother Oncol.

[25] Li Y, Zhao L, Li XF (2021). Hypoxia and the Tumor Microenvironment. Technol Cancer Res Treat.

[26] Schofield CJ, Ratcliffe PJ (2004). Oxygen sensing by HIF hydroxylases. Nat Rev Mol Cell Biol.

[27] Seagroves TN, Ryan HE, Lu H, Wouters BG, Knapp M, Thibault P (2001). Transcription factor HIF-1 is a necessary mediator of the pasteur effect in mammalian cells. Mol Cell Biol.

[28] Shigeta K, Hasegawa M, Hishiki T, Naito Y, Baba Y, Mikami S (2023). IDH2 stabilizes HIF-1α-induced metabolic reprogramming and promotes chemoresistance in urothelial cancer. Embo j.

[29] Wozny AS, Gauthier A, Alphonse G, Malésys C, Varoclier V, Beuve M et al. Involvement of HIF-1α in the Detection, Signaling, and Repair of DNA Double-Strand Breaks after Photon and Carbon-Ion Irradiation. Cancers (Basel). 2021;13(15). 10.3390/cancers13153833 PMC8345054 34359734

[30] Choi C, Son A, Lee GH, Shin SW, Park S, Ahn SH (2019). Targeting DNA-dependent protein kinase sensitizes hepatocellular carcinoma cells to proton beam irradiation through apoptosis induction. PLoS ONE.

[31] Iliakis G, Mladenov E, Mladenova V. Necessities in the Processing of DNA double strand breaks and their effects on genomic instability and Cancer. Cancers (Basel). 2019;11(11). 10.3390/cancers11111671 PMC6896103 31661831

[32] Jiang K, Yin X, Zhang Q, Yin J, Tang Q, Xu M (2023). STC2 activates PRMT5 to induce radioresistance through DNA damage repair and ferroptosis pathways in esophageal squamous cell carcinoma. Redox Biol.

[33] Dengler F. Activation of AMPK under Hypoxia: many roads leading to Rome. Int J Mol Sci. 2020;21(7). 10.3390/ijms21072428 PMC7177550 32244507

[34] Murata Y, Hashimoto T, Urushihara Y, Shiga S, Takeda K, Jingu K (2018). Knockdown of AMPKα decreases ATM expression and increases radiosensitivity under hypoxia and nutrient starvation in an SV40-transformed human fibroblast cell line, LM217. Biochem Biophys Res Commun.

[35] Shiga S, Murata Y, Hashimoto T, Urushihara Y, Fujishima Y, Kudo K (2020). DNA-PKcs is activated under nutrient starvation and activates akt, MST1, FoxO3a, and NDR1. Biochem Biophys Res Commun.

[36] Hashimoto T, Murata Y, Urushihara Y, Shiga S, Takeda K, Hosoi Y (2018). Severe hypoxia increases expression of ATM and DNA-PKcs and it increases their activities through Src and AMPK signaling pathways. Biochem Biophys Res Commun.

[37] Wang D, Li X, Jiao D, Cai Y, Qian L, Shen Y (2023). LCN2 secreted by tissue-infiltrating neutrophils induces the ferroptosis and wasting of adipose and muscle tissues in lung cancer cachexia. J Hematol Oncol.

[38] Chi Y, Remsik J, Kiseliovas V, Derderian C, Sener U, Alghader M (2020). Cancer cells deploy lipocalin-2 to collect limiting iron in leptomeningeal metastasis. Science.

[39] Zhang MX, Wang L, Zeng L, Tu ZW (2020). LCN2 is a potential biomarker for Radioresistance and Recurrence in Nasopharyngeal Carcinoma. Front Oncol.

[40] Chen F, Xu B, Li J, Yang X, Gu J, Yao X (2021). Hypoxic tumour cell-derived exosomal mir-340-5p promotes radioresistance of oesophageal squamous cell carcinoma via KLF10. J Exp Clin Cancer Res.

[41] Bhatt AN, Chauhan A, Khanna S, Rai Y, Singh S, Soni R (2015). Transient elevation of glycolysis confers radio-resistance by facilitating DNA repair in cells. BMC Cancer.

[42] Hao J, Graham P, Chang L, Ni J, Wasinger V, Beretov J (2016). Proteomic identification of the lactate dehydrogenase A in a radioresistant prostate cancer xenograft mouse model for improving radiotherapy. Oncotarget.

[43] Huang TQ, Bi YN, Cui Z, Guan JP, Huang YC (2020). MUC1 confers radioresistance in head and neck squamous cell carcinoma (HNSCC) cells. Bioengineered.

[44] Fu X, Tang N, Xie WQ, Mao L, Qiu YD (2020). MUC1 promotes glycolysis through inhibiting BRCA1 expression in pancreatic cancer. Chin J Nat Med.

[45] Gunda V, Souchek J, Abrego J, Shukla SK, Goode GD, Vernucci E (2017). MUC1-Mediated metabolic alterations regulate response to Radiotherapy in Pancreatic Cancer. Clin Cancer Res.

[46] Jia C, Zhao Y, Huang H, Fan K, Xie T, Xie M (2022). Apigenin sensitizes radiotherapy of mouse subcutaneous glioma through attenuations of cell stemness and DNA damage repair by inhibiting NF-κB/HIF-1α-mediated glycolysis. J Nutr Biochem.

[47] Fujimoto M, Takii R, Takaki E, Katiyar A, Nakato R, Shirahige K (2017). The HSF1-PARP13-PARP1 complex facilitates DNA repair and promotes mammary tumorigenesis. Nat Commun.

[48] Li Q, Martinez JD (2011). Loss of HSF1 results in defective radiation-induced G(2) arrest and DNA repair. Radiat Res.

[49] He L, Lv S, Ma X, Jiang S, Zhou F, Zhang Y (2022). ErbB2 promotes breast cancer metastatic potential via HSF1/LDHA axis-mediated glycolysis. Med Oncol.

[50] Wu S, Cao R, Tao B, Wu P, Peng C, Gao H (2022). Pyruvate facilitates FACT-Mediated γH2AX loading to Chromatin and promotes the Radiation Resistance of Glioblastoma. Adv Sci (Weinh).

[51] Wang JZ, Zhu W, Han J, Yang X, Zhou R, Lu HC (2021). The role of the HIF-1α/ALYREF/PKM2 axis in glycolysis and tumorigenesis of bladder cancer. Cancer Commun (Lond).

[52] Klein C, Dokic I, Mairani A, Mein S, Brons S, Häring P (2017). Overcoming hypoxia-induced tumor radioresistance in non-small cell lung cancer by targeting DNA-dependent protein kinase in combination with carbon ion irradiation. Radiat Oncol.

[53] Wozny AS, Alphonse G, Cassard A, Malésys C, Louati S, Beuve M (2020). Impact of hypoxia on the double-strand break repair after photon and carbon ion irradiation of radioresistant HNSCC cells. Sci Rep.

[54] Cho RW, Clarke MF (2008). Recent advances in cancer stem cells. Curr Opin Genet Dev.

[55] Al-Hajj M, Wicha MS, Benito-Hernandez A, Morrison SJ, Clarke MF (2003). Prospective identification of tumorigenic breast cancer cells. Proc Natl Acad Sci U S A.

[56] Hanahan D, Weinberg RA (2011). Hallmarks of cancer: the next generation. Cell.

[57] Su S, Chen J, Yao H, Liu J, Yu S, Lao L (2018). CD10(+)GPR77(+) Cancer-Associated fibroblasts promote Cancer formation and chemoresistance by sustaining Cancer Stemness. Cell.

[58] Qureshi-Baig K, Kuhn D, Viry E, Pozdeev VI, Schmitz M, Rodriguez F (2020). Hypoxia-induced autophagy drives colorectal cancer initiation and progression by activating the PRKC/PKC-EZR (ezrin) pathway. Autophagy.

[59] Ramteke A, Ting H, Agarwal C, Mateen S, Somasagara R, Hussain A (2015). Exosomes secreted under hypoxia enhance invasiveness and stemness of prostate cancer cells by targeting adherens junction molecules. Mol Carcinog.

[60] Creighton CJ, Li X, Landis M, Dixon JM, Neumeister VM, Sjolund A (2009). Residual breast cancers after conventional therapy display mesenchymal as well as tumor-initiating features. Proc Natl Acad Sci U S A.

[61] Zhu L, Gibson P, Currle DS, Tong Y, Richardson RJ, Bayazitov IT (2009). Prominin 1 marks intestinal stem cells that are susceptible to neoplastic transformation. Nature.

[62] Kabakov A, Yakimova A, Matchuk O. Molecular chaperones in Cancer Stem cells: determinants of stemness and potential targets for Antitumor Therapy. Cells. 2020;9(4). 10.3390/cells9040892 PMC7226806 32268506

[63] Phillips TM, McBride WH, Pajonk F (2006). The response of CD24(-/low)/CD44 + breast cancer-initiating cells to radiation. J Natl Cancer Inst.

[64] Nascimento-Filho CHV, Webber LP, Borgato GB, Goloni-Bertollo EM, Squarize CH, Castilho RM (2019). Hypoxic niches are endowed with a protumorigenic mechanism that supersedes the protective function of PTEN. Faseb j.

[65] Wang M, Li X, Qu Y, Xu O, Sun Q (2013). Hypoxia promotes radioresistance of CD133-positive Hep-2 human laryngeal squamous carcinoma cells in vitro. Int J Oncol.

[66] Balamurugan K, Mendoza-Villanueva D, Sharan S, Summers GH, Dobrolecki LE, Lewis MT (2019). C/EBPδ links IL-6 and HIF-1 signaling to promote breast cancer stem cell-associated phenotypes. Oncogene.

[67] Beck B, Driessens G, Goossens S, Youssef KK, Kuchnio A, Caauwe A (2011). A vascular niche and a VEGF-Nrp1 loop regulate the initiation and stemness of skin tumours. Nature.

[68] Loda A, Calza S, Giacomini A, Ravelli C, Krishna Chandran AM, Tobia C (2023). FGF-trapping hampers cancer stem-like cells in uveal melanoma. Cancer Cell Int.

[69] Xiong H, Nie X, Zou Y, Gong C, Li Y, Wu H (2017). Twist1 enhances Hypoxia Induced Radioresistance in Cervical Cancer cells by promoting Nuclear EGFR localization. J Cancer.

[70] Yao T, Weng X, Yao Y, Huang C, Li J, Peng Y (2020). ALDH-1-positive cells exhibited a radioresistant phenotype that was enhanced with hypoxia in cervical cancer. BMC Cancer.

[71] Kim IG, Lee JH, Kim SY, Hwang HM, Kim TR, Cho EW (2018). Hypoxia-inducible transgelin 2 selects epithelial-to-mesenchymal transition and γ-radiation-resistant subtypes by focal adhesion kinase-associated insulin-like growth factor 1 receptor activation in non-small-cell lung cancer cells. Cancer Sci.

[72] Liang DH, El-Zein R, Dave B. Autophagy inhibition to increase radiosensitization in breast Cancer. J Nucl Med Radiat Ther. 2015;6(5). 10.4172/2155-9619.1000254 PMC4657142 26613064

[73] Wozny AS, Lauret A, Battiston-Montagne P, Guy JB, Beuve M, Cunha M (2017). Differential pattern of HIF-1α expression in HNSCC cancer stem cells after carbon ion or photon irradiation: one molecular explanation of the oxygen effect. Br J Cancer.

[74] Cui CP, Wong CC, Kai AK, Ho DW, Lau EY, Tsui YM (2017). SENP1 promotes hypoxia-induced cancer stemness by HIF-1α deSUMOylation and SENP1/HIF-1α positive feedback loop. Gut.

[75] Yang W, Wei J, Guo T, Shen Y, Liu F (2014). Knockdown of miR-210 decreases hypoxic glioma stem cells stemness and radioresistance. Exp Cell Res.

[76] Chen X, Wu L, Li D, Xu Y, Zhang L, Niu K (2018). Radiosensitizing effects of miR-18a-5p on lung cancer stem-like cells via downregulating both ATM and HIF-1α. Cancer Med.

[77] Wu SL, Li YJ, Liao K, Shi L, Zhang N, Liu S (2017). 2-Methoxyestradiol inhibits the proliferation and migration and reduces the radioresistance of nasopharyngeal carcinoma CNE-2 stem cells via NF-κB/HIF-1 signaling pathway inactivation and EMT reversal. Oncol Rep.

[78] Piao HY, Guo S, Wang Y, Zhang J (2021). Exosome-transmitted lncRNA PCGEM1 promotes invasive and metastasis in gastric cancer by maintaining the stability of SNAI1. Clin Transl Oncol.

[79] Jin H, Kim HJ. NK cells lose their cytotoxicity function against Cancer Stem Cell-Rich Radiotherapy-resistant breast Cancer cell populations. Int J Mol Sci. 2021;22(17). 10.3390/ijms22179639 PMC8431804 34502547

[80] Salimian Rizi B, Caneba C, Nowicka A, Nabiyar AW, Liu X, Chen K (2015). Nitric oxide mediates metabolic coupling of omentum-derived adipose stroma to ovarian and endometrial cancer cells. Cancer Res.

[81] Shen YA, Wang CY, Hsieh YT, Chen YJ, Wei YH (2015). Metabolic reprogramming orchestrates cancer stem cell properties in nasopharyngeal carcinoma. Cell Cycle.

[82] Song K, Kwon H, Han C, Zhang J, Dash S, Lim K (2015). Active glycolytic metabolism in CD133(+) hepatocellular cancer stem cells: regulation by MIR-122. Oncotarget.

[83] Chen CL, Uthaya Kumar DB, Punj V, Xu J, Sher L, Tahara SM (2016). NANOG metabolically reprograms Tumor-initiating stem-like cells through tumorigenic changes in oxidative phosphorylation and fatty acid metabolism. Cell Metab.

[84] Liu PP, Liao J, Tang ZJ, Wu WJ, Yang J, Zeng ZL (2014). Metabolic regulation of cancer cell side population by glucose through activation of the akt pathway. Cell Death Differ.

[85] Bi L, Ren Y, Feng M, Meng P, Wang Q, Chen W (2021). HDAC11 regulates Glycolysis through the LKB1/AMPK Signaling Pathway to maintain Hepatocellular Carcinoma Stemness. Cancer Res.

[86] Palorini R, Votta G, Balestrieri C, Monestiroli A, Olivieri S, Vento R (2014). Energy metabolism characterization of a novel cancer stem cell-like line 3AB-OS. J Cell Biochem.

[87] Sun L, Moritake T, Ito K, Matsumoto Y, Yasui H, Nakagawa H (2017). Metabolic analysis of radioresistant medulloblastoma stem-like clones and potential therapeutic targets. PLoS ONE.

[88] Zhao Y, Huang H, Jia CH, Fan K, Xie T, Zhu ZY (2021). Apigenin increases radiosensitivity of glioma stem cells by attenuating HIF-1α-mediated glycolysis. Med Oncol.

[89] Mao P, Joshi K, Li J, Kim SH, Li P, Santana-Santos L (2013). Mesenchymal glioma stem cells are maintained by activated glycolytic metabolism involving aldehyde dehydrogenase 1A3. Proc Natl Acad Sci U S A.

[90] Lettini G, Sisinni L, Condelli V, Matassa DS, Simeon V, Maddalena F (2016). TRAP1 regulates stemness through Wnt/β-catenin pathway in human colorectal carcinoma. Cell Death Differ.

[91] Yoshida S, Tsutsumi S, Muhlebach G, Sourbier C, Lee MJ, Lee S (2013). Molecular chaperone TRAP1 regulates a metabolic switch between mitochondrial respiration and aerobic glycolysis. Proc Natl Acad Sci U S A.

[92] Yu Y, Du H, Wei S, Feng L, Li J, Yao F (2018). Adipocyte-derived exosomal MiR-27a induces insulin resistance in skeletal muscle through repression of PPARγ. Theranostics.

[93] Luo X, Zheng E, Wei L, Zeng H, Qin H, Zhang X (2021). The fatty acid receptor CD36 promotes HCC progression through activating Src/PI3K/AKT axis-dependent aerobic glycolysis. Cell Death Dis.

[94] Yang CF, Yang GD, Huang TJ, Li R, Chu QQ, Xu L (2016). EB-virus latent membrane protein 1 potentiates the stemness of nasopharyngeal carcinoma via preferential activation of PI3K/AKT pathway by a positive feedback loop. Oncogene.

[95] Olivares-Urbano MA, Griñán-Lisón C, Marchal JA, Núñez MI. CSC Radioresistance: a therapeutic challenge to improve Radiotherapy Effectiveness in Cancer. Cells. 2020;9(7). 10.3390/cells9071651 PMC7407195 32660072

[96] Gu D, Liu H, Su GH, Zhang X, Chin-Sinex H, Hanenberg H (2013). Combining hedgehog signaling inhibition with focal irradiation on reduction of pancreatic cancer metastasis. Mol Cancer Ther.

[97] Liu Y, Yang M, Luo J, Zhou H (2020). Radiotherapy targeting cancer stem cells awakens them to induce tumour relapse and metastasis in oral cancer. Int J Oral Sci.

[98] Matchuk ON, Orlova NV, Zamulaeva IA (2016). Changes in the relative number of SP cells of Melanoma Line B16 after Radiation exposure in vivo. Radiats Biol Radioecol.

[99] Narang H, Kumar A, Bhat N, Pandey BN, Ghosh A (2015). Effect of proton and gamma irradiation on human lung carcinoma cells: gene expression, cell cycle, cell death, epithelial-mesenchymal transition and cancer-stem cell trait as biological end points. Mutat Res.

[100] Chiblak S, Tang Z, Lemke D, Knoll M, Dokic I, Warta R et al. Carbon irradiation overcomes glioma radioresistance by eradicating stem cells and forming an antiangiogenic and immunopermissive niche. JCI Insight. 2019;4(2). 10.1172/jci.insight.123837 PMC6413779 30674721

[101] Hu Y, Rosen DG, Zhou Y, Feng L, Yang G, Liu J (2005). Mitochondrial manganese-superoxide dismutase expression in ovarian cancer: role in cell proliferation and response to oxidative stress. J Biol Chem.

[102] Young TW, Mei FC, Yang G, Thompson-Lanza JA, Liu J, Cheng X (2004). Activation of antioxidant pathways in ras-mediated oncogenic transformation of human surface ovarian epithelial cells revealed by functional proteomics and mass spectrometry. Cancer Res.

[103] Hsieh CH, Shyu WC, Chiang CY, Kuo JW, Shen WC, Liu RS (2011). NADPH oxidase subunit 4-mediated reactive oxygen species contribute to cycling hypoxia-promoted tumor progression in glioblastoma multiforme. PLoS ONE.

[104] Song B, Zhang Q, Yu M, Qi X, Wang G, Xiao L (2017). Ursolic acid sensitizes radioresistant NSCLC cells expressing HIF-1α through reducing endogenous GSH and inhibiting HIF-1α. Oncol Lett.

[105] Hayes JD, McLellan LI (1999). Glutathione and glutathione-dependent enzymes represent a co-ordinately regulated defence against oxidative stress. Free Radic Res.

[106] Zou P, Xia Y, Ji J, Chen W, Zhang J, Chen X (2016). Piperlongumine as a direct TrxR1 inhibitor with suppressive activity against gastric cancer. Cancer Lett.

[107] Vukovic V, Nicklee T, Hedley DW (2001). Differential effects of buthionine sulphoximine in hypoxic and non-hypoxic regions of human cervical carcinoma xenografts. Radiother Oncol.

[108] Wang H, Bouzakoura S, de Mey S, Jiang H, Law K, Dufait I (2017). Auranofin radiosensitizes tumor cells through targeting thioredoxin reductase and resulting overproduction of reactive oxygen species. Oncotarget.

[109] Xia C, Meng Q, Liu LZ, Rojanasakul Y, Wang XR, Jiang BH (2007). Reactive oxygen species regulate angiogenesis and tumor growth through vascular endothelial growth factor. Cancer Res.

[110] Marcone S, Buckley A, Ryan CJ, McCabe M, Lynam-Lennon N, Matallanas D (2021). Proteomic signatures of radioresistance: alteration of inflammation, angiogenesis and metabolism-related factors in radioresistant oesophageal adenocarcinoma. Cancer Treat Res Commun.

[111] Shin E, Kim B, Kang H, Lee H, Park J, Kang J (2023). Mitochondrial glutamate transporter SLC25A22 uni-directionally export glutamate for metabolic rewiring in radioresistant glioblastoma. Int J Biol Macromol.

[112] Wen J, Xiong K, Aili A, Wang H, Zhu Y, Yu Z (2020). PEX5, a novel target of microRNA-31-5p, increases radioresistance in hepatocellular carcinoma by activating Wnt/β-catenin signaling and homologous recombination. Theranostics.

[113] Li P, Zhang D, Shen L, Dong K, Wu M, Ou Z (2016). Redox homeostasis protects mitochondria through accelerating ROS conversion to enhance hypoxia resistance in cancer cells. Sci Rep.

[114] Xue C, Li X, Liu G, Liu W (2016). Evaluation of mitochondrial respiratory chain on the generation of reactive oxygen species and cytotoxicity in HaCaT cells Induced by Nanosized Titanium Dioxide under UVA Irradiation. Int J Toxicol.

[115] Iatsenko I, Boquete JP, Lemaitre B (2018). Microbiota-Derived Lactate activates production of reactive oxygen species by the Intestinal NADPH Oxidase Nox and Shortens Drosophila Lifespan. Immunity.

[116] Diepart C, Karroum O, Magat J, Feron O, Verrax J, Calderon PB (2012). Arsenic trioxide treatment decreases the oxygen consumption rate of tumor cells and radiosensitizes solid tumors. Cancer Res.

[117] Levy JMM, Towers CG, Thorburn A (2017). Targeting autophagy in cancer. Nat Rev Cancer.

[118] Sharif T, Martell E, Dai C, Kennedy BE, Murphy P, Clements DR (2017). Autophagic homeostasis is required for the pluripotency of cancer stem cells. Autophagy.

[119] Keulers TG, Koch A, van Gisbergen MW, Barbeau LMO, Zonneveld MI, de Jong MC (2022). ATG12 deficiency results in intracellular glutamine depletion, abrogation of tumor hypoxia and a favorable prognosis in cancer. Autophagy.

[120] He WS, Dai XF, Jin M, Liu CW, Rent JH (2012). Hypoxia-induced autophagy confers resistance of breast cancer cells to ionizing radiation. Oncol Res.

[121] Zhong R, Xu H, Chen G, Zhao G, Gao Y, Liu X (2015). The role of hypoxia-inducible factor-1α in radiation-induced autophagic cell death in breast cancer cells. Tumour Biol.

[122] Chung SJ, Nagaraju GP, Nagalingam A, Muniraj N, Kuppusamy P, Walker A (2017). ADIPOQ/adiponectin induces cytotoxic autophagy in breast cancer cells through STK11/LKB1-mediated activation of the AMPK-ULK1 axis. Autophagy.

[123] Chhipa RR, Fan Q, Anderson J, Muraleedharan R, Huang Y, Ciraolo G (2018). AMP kinase promotes glioblastoma bioenergetics and tumour growth. Nat Cell Biol.

[124] Lin L, Huang H, Liao W, Ma H, Liu J, Wang L (2015). MACC1 supports human gastric cancer growth under metabolic stress by enhancing the Warburg effect. Oncogene.

[125] Hardie DG (2015). AMPK: positive and negative regulation, and its role in whole-body energy homeostasis. Curr Opin Cell Biol.

[126] Chaube B, Malvi P, Singh SV, Mohammad N, Viollet B, Bhat MK (2015). AMPK maintains energy homeostasis and survival in cancer cells via regulating p38/PGC-1α-mediated mitochondrial biogenesis. Cell Death Discov.

[127] Sun W, Jia M, Feng Y, Cheng X (2023). Lactate is a bridge linking glycolysis and autophagy through lactylation. Autophagy.

[128] Abdel-Rafei MK, Thabet NM, Rashed LA, Moustafa EM (2021). Canagliflozin, a SGLT-2 inhibitor, relieves ER stress, modulates autophagy and induces apoptosis in irradiated HepG2 cells: Signal transduction between PI3K/AKT/GSK-3β/mTOR and Wnt/β-catenin pathways; in vitro. J Cancer Res Ther.

[129] Shen Y, Liu Y, Sun T, Yang W (2017). LincRNA-p21 knockdown enhances radiosensitivity of hypoxic tumor cells by reducing autophagy through HIF-1/Akt/mTOR/P70S6K pathway. Exp Cell Res.

[130] Nagelkerke A, Bussink J, van der Kogel AJ, Sweep FC, Span PN (2013). The PERK/ATF4/LAMP3-arm of the unfolded protein response affects radioresistance by interfering with the DNA damage response. Radiother Oncol.

[131] Zheng R, Yao Q, Xie G, Du S, Ren C, Wang Y (2015). TAT-ODD-p53 enhances the radiosensitivity of hypoxic breast cancer cells by inhibiting parkin-mediated mitophagy. Oncotarget.

[132] Chen X, Wang P, Guo F, Wang X, Wang J, Xu J (2017). Autophagy enhanced the radioresistance of non-small cell lung cancer by regulating ROS level under hypoxia condition. Int J Radiat Biol.

[133] Zou YM, Hu GY, Zhao XQ, Lu T, Zhu F, Yu SY (2014). Hypoxia-induced autophagy contributes to radioresistance via c-Jun-mediated Beclin1 expression in lung cancer cells. J Huazhong Univ Sci Technolog Med Sci.

[134] Sun Y, Xing X, Liu Q, Wang Z, Xin Y, Zhang P (2015). Hypoxia-induced autophagy reduces radiosensitivity by the HIF-1α/miR-210/Bcl-2 pathway in colon cancer cells. Int J Oncol.

[135] Gu H, Liu M, Ding C, Wang X, Wang R, Wu X (2016). Hypoxia-responsive miR-124 and miR-144 reduce hypoxia-induced autophagy and enhance radiosensitivity of prostate cancer cells via suppressing PIM1. Cancer Med.

[136] Wang W, Liu M, Guan Y, Wu Q (2016). Hypoxia-responsive Mir-301a and Mir-301b promote Radioresistance of prostate Cancer cells via downregulating NDRG2. Med Sci Monit.

[137] Zhao M, Zhang Y, Jiang Y, Wang K, Wang X, Zhou D (2021). YAP promotes autophagy and progression of gliomas via upregulating HMGB1. J Exp Clin Cancer Res.

[138] Rouschop KM, van den Beucken T, Dubois L, Niessen H, Bussink J, Savelkouls K (2010). The unfolded protein response protects human tumor cells during hypoxia through regulation of the autophagy genes MAP1LC3B and ATG5. J Clin Invest.

[139] Moergel M, Abt E, Stockinger M, Kunkel M (2010). Overexpression of p63 is associated with radiation resistance and prognosis in oral squamous cell carcinoma. Oral Oncol.

[140] Liu H, Zheng W, Chen Q, Zhou Y, Pan Y, Zhang J et al. lncRNA CASC19 contributes to Radioresistance of Nasopharyngeal Carcinoma by promoting Autophagy via AMPK-mTOR pathway. Int J Mol Sci. 2021;22(3). 10.3390/ijms22031407 PMC7866785 33573349

[141] Tsuboi Y, Kurimoto M, Nagai S, Hayakawa Y, Kamiyama H, Hayashi N (2009). Induction of autophagic cell death and radiosensitization by the pharmacological inhibition of nuclear factor-kappa B activation in human glioma cell lines. J Neurosurg.

[142] Song L, Liu S, Zhang L, Yao H, Gao F, Xu D (2016). MiR-21 modulates radiosensitivity of cervical cancer through inhibiting autophagy via the PTEN/Akt/HIF-1α feedback loop and the Akt-mTOR signaling pathway. Tumour Biol.

[143] Classen F, Kranz P, Riffkin H, Pompsch M, Wolf A, Göpelt K (2019). Autophagy induced by ionizing radiation promotes cell death over survival in human colorectal cancer cells. Exp Cell Res.

[144] Liu B, Han D, Zhang T, Cheng G, Lu Y, Wang J (2019). Hypoxia-induced autophagy promotes EGFR loss in specific cell contexts, which leads to cell death and enhanced radiosensitivity. Int J Biochem Cell Biol.

[145] Kuger S, Cörek E, Polat B, Kämmerer U, Flentje M, Djuzenova CS (2014). Novel PI3K and mTOR inhibitor NVP-BEZ235 radiosensitizes breast Cancer cell lines under normoxic and hypoxic conditions. Breast Cancer (Auckl).

[146] Cao J, Liu X, Yang Y, Wei B, Li Q, Mao G (2020). Decylubiquinone suppresses breast cancer growth and metastasis by inhibiting angiogenesis via the ROS/p53/ BAI1 signaling pathway. Angiogenesis.

[147] Kanugula AK, Adapala RK, Jamaiyar A, Lenkey N, Guarino BD, Liedtke W (2021). Endothelial TRPV4 channels prevent tumor growth and metastasis via modulation of tumor angiogenesis and vascular integrity. Angiogenesis.

[148] Peterson H. Tumor blood circulation: angiogenesis, vascular morphology, and blood flow of experimental and human tumors. 1979.

[149] Wachsberger PR, Burd R, Marero N, Daskalakis C, Ryan A, McCue P (2005). Effect of the tumor vascular-damaging agent, ZD6126, on the radioresponse of U87 glioblastoma. Clin Cancer Res.

[150] De Bock K, De Smet F, Leite De Oliveira R, Anthonis K, Carmeliet P (2009). Endothelial oxygen sensors regulate tumor vessel abnormalization by instructing phalanx endothelial cells. J Mol Med (Berl).

[151] Jain RK (2005). Normalization of tumor vasculature: an emerging concept in antiangiogenic therapy. Science.

[152] Griffin RJ, Williams BW, Wild R, Cherrington JM, Park H, Song CW (2002). Simultaneous inhibition of the receptor kinase activity of vascular endothelial, fibroblast, and platelet-derived growth factors suppresses tumor growth and enhances tumor radiation response. Cancer Res.

[153] Shweiki D, Itin A, Soffer D, Keshet E (1992). Vascular endothelial growth factor induced by hypoxia may mediate hypoxia-initiated angiogenesis. Nature.

[154] Guleng B, Han J, Yang JQ, Huang QW, Huang JK, Yang XN (2012). TFF3 mediated induction of VEGF via hypoxia in human gastric cancer SGC-7901 cells. Mol Biol Rep.

[155] Zimna A, Kurpisz M (2015). Hypoxia-inducible Factor-1 in physiological and pathophysiological angiogenesis: applications and therapies. Biomed Res Int.

[156] Wang GL, Jiang BH, Rue EA, Semenza GL (1995). Hypoxia-inducible factor 1 is a basic-helix-loop-helix-PAS heterodimer regulated by cellular O2 tension. Proc Natl Acad Sci U S A.

[157] Griffioen AW, Bischoff J (2019). Oxygen sensing decoded: a Nobel concept in biology. Angiogenesis.

[158] Ley CD, Olsen MW, Lund EL, Kristjansen PE (2004). Angiogenic synergy of bFGF and VEGF is antagonized by Angiopoietin-2 in a modified in vivo Matrigel assay. Microvasc Res.

[159] Maisonpierre PC, Suri C, Jones PF, Bartunkova S, Wiegand SJ, Radziejewski C (1997). Angiopoietin-2, a natural antagonist for Tie2 that disrupts in vivo angiogenesis. Science.

[160] Post S, Peeters W, Busser E, Lamers D, Sluijter JP, Goumans MJ (2008). Balance between angiopoietin-1 and angiopoietin-2 is in favor of angiopoietin-2 in atherosclerotic plaques with high microvessel density. J Vasc Res.

[161] Shin SW, Jung W, Choi C, Kim SY, Son A, Kim H et al. Fucoidan-Manganese Dioxide nanoparticles Potentiate Radiation Therapy by co-targeting Tumor Hypoxia and Angiogenesis. Mar Drugs. 2018;16(12). 10.3390/md16120510 PMC6316049 30558324

[162] Yang L, Liu L, Xu Z, Liao W, Feng D, Dong X (2015). EBV-LMP1 targeted DNAzyme enhances radiosensitivity by inhibiting tumor angiogenesis via the JNKs/HIF-1 pathway in nasopharyngeal carcinoma. Oncotarget.

[163] Kruser TJ, Wheeler DL, Armstrong EA, Iida M, Kozak KR, van der Kogel AJ (2010). Augmentation of radiation response by motesanib, a multikinase inhibitor that targets vascular endothelial growth factor receptors. Clin Cancer Res.

[164] Tong RT, Boucher Y, Kozin SV, Winkler F, Hicklin DJ, Jain RK (2004). Vascular normalization by vascular endothelial growth factor receptor 2 blockade induces a pressure gradient across the vasculature and improves drug penetration in tumors. Cancer Res.

[165] Cantelmo AR, Conradi LC, Brajic A, Goveia J, Kalucka J, Pircher A (2016). Inhibition of the glycolytic activator PFKFB3 in Endothelium induces Tumor Vessel normalization, Impairs Metastasis, and improves chemotherapy. Cancer Cell.

[166] Bagley RG, Rouleau C, Morgenbesser SD, Weber W, Cook BP, Shankara S (2006). Pericytes from human non-small cell lung carcinomas: an attractive target for anti-angiogenic therapy. Microvasc Res.

[167] Erber R, Thurnher A, Katsen AD, Groth G, Kerger H, Hammes HP (2004). Combined inhibition of VEGF and PDGF signaling enforces tumor vessel regression by interfering with pericyte-mediated endothelial cell survival mechanisms. Faseb j.

[168] Meng YM, Jiang X, Zhao X, Meng Q, Wu S, Chen Y (2021). Hexokinase 2-driven glycolysis in pericytes activates their contractility leading to tumor blood vessel abnormalities. Nat Commun.

[169] Giatromanolaki A, Sivridis E, Gatter KC, Turley H, Harris AL, Koukourakis MI (2006). Lactate dehydrogenase 5 (LDH-5) expression in endometrial cancer relates to the activated VEGF/VEGFR2(KDR) pathway and prognosis. Gynecol Oncol.

[170] Wachsberger P, Burd R, Dicker AP (2003). Tumor response to ionizing radiation combined with antiangiogenesis or vascular targeting agents: exploring mechanisms of interaction. Clin Cancer Res.

[171] Hlushchuk R, Riesterer O, Baum O, Wood J, Gruber G, Pruschy M (2008). Tumor recovery by angiogenic switch from sprouting to intussusceptive angiogenesis after treatment with PTK787/ZK222584 or ionizing radiation. Am J Pathol.

[172] Jarosz-Biej M, Smolarczyk R, Cichoń T, Kułach N. Tumor Microenvironment as a game changer in Cancer Radiotherapy. Int J Mol Sci. 2019;20(13). 10.3390/ijms20133212 PMC6650939 31261963

[173] Ahn GO, Tseng D, Liao CH, Dorie MJ, Czechowicz A, Brown JM (2010). Inhibition of Mac-1 (CD11b/CD18) enhances tumor response to radiation by reducing myeloid cell recruitment. Proc Natl Acad Sci U S A.

[174] Chiang CS, Fu SY, Wang SC, Yu CF, Chen FH, Lin CM (2012). Irradiation promotes an m2 macrophage phenotype in tumor hypoxia. Front Oncol.

[175] Crittenden MR, Cottam B, Savage T, Nguyen C, Newell P, Gough MJ (2012). Expression of NF-κB p50 in tumor stroma limits the control of tumors by radiation therapy. PLoS ONE.

[176] Kachikwu EL, Iwamoto KS, Liao YP, DeMarco JJ, Agazaryan N, Economou JS (2011). Radiation enhances regulatory T cell representation. Int J Radiat Oncol Biol Phys.

[177] Knitz MW, Bickett TE, Darragh LB, Oweida AJ, Bhatia S, Van Court B et al. Targeting resistance to radiation-immunotherapy in cold HNSCCs by modulating the Treg-dendritic cell axis. J Immunother Cancer. 2021;9(4). 10.1136/jitc-2020-001955 PMC8061827 33883256

[178] Vanpouille-Box C, Diamond JM, Pilones KA, Zavadil J, Babb JS, Formenti SC (2015). TGFβ is a Master Regulator of Radiation Therapy-Induced Antitumor Immunity. Cancer Res.

[179] Kalbasi A, Komar C, Tooker GM, Liu M, Lee JW, Gladney WL (2017). Tumor-derived CCL2 mediates resistance to Radiotherapy in Pancreatic Ductal Adenocarcinoma. Clin Cancer Res.

[180] Yi M, Niu M, Xu L, Luo S, Wu K (2021). Regulation of PD-L1 expression in the tumor microenvironment. J Hematol Oncol.

[181] Deng L, Liang H, Burnette B, Weicheslbaum RR, Fu YX (2014). Radiation and anti-PD-L1 antibody combinatorial therapy induces T cell-mediated depletion of myeloid-derived suppressor cells and tumor regression. Oncoimmunology.

[182] Lee YS, Heo W, Choi HJ, Cho HR, Nam JH, Ki YG (2021). An inhibitor of programmed death ligand 1 enhances natural killer cell-mediated immunity against malignant melanoma cells. PLoS ONE.

[183] Koukourakis IM, Giakzidis AG, Kouroupi M, Giatromanolaki A, Abatzoglou I, Karpouzis A (2021). Cutaneous squamous-cell carcinoma of the head-neck area refractory to chemo-radiotherapy: benefit from anti-PD-1 immunotherapy. BJR Case Rep.

[184] Zhang J, Bu X, Wang H, Zhu Y, Geng Y, Nihira NT (2018). Cyclin D-CDK4 kinase destabilizes PD-L1 via cullin 3-SPOP to control cancer immune surveillance. Nature.

[185] Liu Z, Wang T, She Y, Wu K, Gu S, Li L (2021). N(6)-methyladenosine-modified circIGF2BP3 inhibits CD8(+) T-cell responses to facilitate tumor immune evasion by promoting the deubiquitination of PD-L1 in non-small cell lung cancer. Mol Cancer.

[186] Meyer F, Engel AM, Krause AK, Wagner T, Poole L, Dubrovska A (2022). Efficient DNA repair mitigates replication stress resulting in less immunogenic cytosolic DNA in radioresistant breast Cancer stem cells. Front Immunol.

[187] Petragnano F, Pietrantoni I, Camero S, Codenotti S, Milazzo L, Vulcano F (2020). Clinically relevant radioresistant rhabdomyosarcoma cell lines: functional, molecular and immune-related characterization. J Biomed Sci.

[188] Ouédraogo ZG, Müller-Barthélémy M, Kemeny JL, Dedieu V, Biau J, Khalil T (2016). STAT3 serine 727 phosphorylation: a relevant Target to Radiosensitize Human Glioblastoma. Brain Pathol.

[189] Pizon M, Schott DS, Pachmann U, Pachmann K (2018). B7-H3 on circulating epithelial tumor cells correlates with the proliferation marker, Ki-67, and may be associated with the aggressiveness of tumors in breast cancer patients. Int J Oncol.

[190] Gottfried E, Kunz-Schughart LA, Ebner S, Mueller-Klieser W, Hoves S, Andreesen R (2006). Tumor-derived lactic acid modulates dendritic cell activation and antigen expression. Blood.

[191] Liang H, Deng L, Hou Y, Meng X, Huang X, Rao E (2017). Host STING-dependent MDSC mobilization drives extrinsic radiation resistance. Nat Commun.

[192] Hou Y, Liang H, Rao E, Zheng W, Huang X, Deng L (2018). Non-canonical NF-κB antagonizes STING sensor-mediated DNA sensing in Radiotherapy. Immunity.

[193] Rodriguez PC, Quiceno DG, Zabaleta J, Ortiz B, Zea AH, Piazuelo MB (2004). Arginase I production in the tumor microenvironment by mature myeloid cells inhibits T-cell receptor expression and antigen-specific T-cell responses. Cancer Res.

[194] Noman MZ, Desantis G, Janji B, Hasmim M, Karray S, Dessen P (2014). PD-L1 is a novel direct target of HIF-1α, and its blockade under hypoxia enhanced MDSC-mediated T cell activation. J Exp Med.

[195] Kim AR, Choi SJ, Park J, Kwon M, Chowdhury T, Yu HJ (2022). Spatial immune heterogeneity of hypoxia-induced exhausted features in high-grade glioma. Oncoimmunology.

[196] Colonna-Romano S, Arnold W, Schlüter A, Boistard P, Pühler A, Priefer UB (1990). An fnr-like protein encoded in Rhizobium leguminosarum biovar viciae shows structural and functional homology to Rhizobium meliloti FixK. Mol Gen Genet.

[197] Corzo CA, Condamine T, Lu L, Cotter MJ, Youn JI, Cheng P (2010). HIF-1α regulates function and differentiation of myeloid-derived suppressor cells in the tumor microenvironment. J Exp Med.

[198] Doedens AL, Stockmann C, Rubinstein MP, Liao D, Zhang N, DeNardo DG (2010). Macrophage expression of hypoxia-inducible factor-1 alpha suppresses T-cell function and promotes tumor progression. Cancer Res.

[199] Jung JE, Lee HG, Cho IH, Chung DH, Yoon SH, Yang YM (2005). STAT3 is a potential modulator of HIF-1-mediated VEGF expression in human renal carcinoma cells. Faseb j.

[200] Zhang Q, Zhang C, He J, Guo Q, Hu D, Yang X (2015). STAT3 inhibitor stattic enhances radiosensitivity in esophageal squamous cell carcinoma. Tumour Biol.

[201] Miska J, Lee-Chang C, Rashidi A, Muroski ME, Chang AL, Lopez-Rosas A (2019). HIF-1α is a metabolic switch between Glycolytic-Driven Migration and oxidative phosphorylation-driven immunosuppression of Tregs in Glioblastoma. Cell Rep.

[202] Singer K, Gottfried E, Kreutz M, Mackensen A (2011). Suppression of T-cell responses by tumor metabolites. Cancer Immunol Immunother.

[203] Gupta S, Dwarakanath BS (2020). Modulation of Immuno-biome during radio-sensitization of tumors by glycolytic inhibitors. Curr Med Chem.

[204] Peng M, Yin N, Chhangawala S, Xu K, Leslie CS, Li MO (2016). Aerobic glycolysis promotes T helper 1 cell differentiation through an epigenetic mechanism. Science.

[205] Xu K, Yin N, Peng M, Stamatiades EG, Shyu A, Li P (2021). Glycolysis fuels phosphoinositide 3-kinase signaling to bolster T cell immunity. Science.

[206] Xia C, Li M, Ran G, Wang X, Lu Z, Li T (2021). Redox-responsive nanoassembly restrained myeloid-derived suppressor cells recruitment through autophagy-involved lactate dehydrogenase a silencing for enhanced cancer immunochemotherapy. J Control Release.

[207] Notarangelo G, Spinelli JB, Perez EM, Baker GJ, Kurmi K, Elia I (2022). Oncometabolite d-2HG alters T cell metabolism to impair CD8(+) T cell function. Science.

[208] Scharping NE, Rivadeneira DB, Menk AV, Vignali PDA, Ford BR, Rittenhouse NL (2021). Mitochondrial stress induced by continuous stimulation under hypoxia rapidly drives T cell exhaustion. Nat Immunol.

[209] Goetze K, Walenta S, Ksiazkiewicz M, Kunz-Schughart LA, Mueller-Klieser W (2011). Lactate enhances motility of tumor cells and inhibits monocyte migration and cytokine release. Int J Oncol.

[210] Dietl K, Renner K, Dettmer K, Timischl B, Eberhart K, Dorn C (2010). Lactic acid and acidification inhibit TNF secretion and glycolysis of human monocytes. J Immunol.

[211] Brand A, Singer K, Koehl GE, Kolitzus M, Schoenhammer G, Thiel A (2016). LDHA-Associated Lactic Acid Production blunts Tumor Immunosurveillance by T and NK Cells. Cell Metab.

[212] Gerriets VA, Kishton RJ, Johnson MO, Cohen S, Siska PJ, Nichols AG (2016). Foxp3 and toll-like receptor signaling balance T(reg) cell anabolic metabolism for suppression. Nat Immunol.

[213] Michalek RD, Gerriets VA, Jacobs SR, Macintyre AN, MacIver NJ, Mason EF (2011). Cutting edge: distinct glycolytic and lipid oxidative metabolic programs are essential for effector and regulatory CD4 + T cell subsets. J Immunol.

[214] Sun C, Liu X, Wang B, Wang Z, Liu Y, Di C (2019). Endocytosis-mediated mitochondrial transplantation: transferring normal human astrocytic mitochondria into glioma cells rescues aerobic respiration and enhances radiosensitivity. Theranostics.

[215] Jin X, Kuang Y, Li L, Li H, Zhao T, He Y (2022). A positive feedback circuit comprising p21 and HIF-1α aggravates hypoxia-induced radioresistance of glioblastoma by promoting Glut1/LDHA-mediated glycolysis. Faseb j.

[216] Palayoor ST, Mitchell JB, Cerna D, Degraff W, John-Aryankalayil M, Coleman CN (2008). PX-478, an inhibitor of hypoxia-inducible factor-1alpha, enhances radiosensitivity of prostate carcinoma cells. Int J Cancer.

[217] Helbig L, Koi L, Brüchner K, Gurtner K, Hess-Stumpp H, Unterschemmann K (2014). Hypoxia-inducible factor pathway inhibition resolves tumor hypoxia and improves local tumor control after single-dose irradiation. Int J Radiat Oncol Biol Phys.

[218] Iijima M, Gombodorj N, Tachibana Y, Tachibana K, Yokobori T, Honma K (2018). Development of single nanometer-sized ultrafine oxygen bubbles to overcome the hypoxia-induced resistance to radiation therapy via the suppression of hypoxia-inducible factor–1α. Int J Oncol.

[219] Digomann D, Linge A, Dubrovska A (2019). SLC3A2/CD98hc, autophagy and tumor radioresistance: a link confirmed. Autophagy.

[220] Mukha A, Kahya U, Dubrovska A (2021). Targeting glutamine metabolism and autophagy: the combination for prostate cancer radiosensitization. Autophagy.

[221] Yang WC, Hsu FM, Yang PC (2020). Precision radiotherapy for non-small cell lung cancer. J Biomed Sci.

[222] Kulinich DP, Sheppard JP, Nguyen T, Kondajji AM, Unterberger A, Duong C (2021). Radiotherapy versus combination radiotherapy-bevacizumab for the treatment of recurrent high-grade glioma: a systematic review. Acta Neurochir (Wien).

[223] Landau BR, Laszlo J, Stengle J, Burk D (1958). Certain metabolic and pharmacologic effects in cancer patients given infusions of 2-deoxy-D-glucose. J Natl Cancer Inst.

[224] Raez LE, Papadopoulos K, Ricart AD, Chiorean EG, Dipaola RS, Stein MN (2013). A phase I dose-escalation trial of 2-deoxy-D-glucose alone or combined with docetaxel in patients with advanced solid tumors. Cancer Chemother Pharmacol.

[225] Mohanti BK, Rath GK, Anantha N, Kannan V, Das BS, Chandramouli BA (1996). Improving cancer radiotherapy with 2-deoxy-D-glucose: phase I/II clinical trials on human cerebral gliomas. Int J Radiat Oncol Biol Phys.

[226] Singh D, Banerji AK, Dwarakanath BS, Tripathi RP, Gupta JP, Mathew TL (2005). Optimizing cancer radiotherapy with 2-deoxy-d-glucose dose escalation studies in patients with glioblastoma multiforme. Strahlenther Onkol.

[227] Halford S, Veal GJ, Wedge SR, Payne GS, Bacon CM, Sloan P (2023). A phase I dose-escalation study of AZD3965, an oral Monocarboxylate transporter 1 inhibitor, in patients with Advanced Cancer. Clin Cancer Res.

[228] Bola BM, Chadwick AL, Michopoulos F, Blount KG, Telfer BA, Williams KJ (2014). Inhibition of monocarboxylate transporter-1 (MCT1) by AZD3965 enhances radiosensitivity by reducing lactate transport. Mol Cancer Ther.

